# Integrative multi-omics stratification and translational evaluation of Treg-targeted combination immunotherapy in breast cancer

**DOI:** 10.3389/fonc.2025.1731411

**Published:** 2026-01-06

**Authors:** Nari Kim, Seongwon Na, Hyo Jin Lee, Woojin Yi, Ga Won Son, Jin Park, Jisung Jang, Mihyun Kim, Seong-Yun Jeong, Kyung Won Kim

**Affiliations:** 1 Biomedical Research Center, Asan Institute for Life Sciences, Asan Medical Center, Seoul, Republic of Korea; 2 Departments of Radiology and Research Institute of Radiology, Asan Medical Center, College of Medicine, University of Ulsan, Seoul, Republic of Korea; 3 Asan Institute for Life Sciences, Asan Medical Center, Seoul, Republic of Korea; 4 Asan Medical Institute of Convergence Science and Technology, Asan Medical Center, University of Ulsan College of Medicine, Seoul, Republic of Korea; 5 Department of Convergence Medicine, ASAN Medical Center, University of Ulsan College of Medicine, Seoul, Republic of Korea; 6 Trial Informatics Inc., Seoul, Republic of Korea

**Keywords:** breast cancer, immunosuppressive tumormicroenvironment, multi-omics, patient stratification, regulatory T cells (Tregs), translational oncology

## Abstract

**Background:**

Immunosuppressive breast cancer subtypes driven by regulatory T cells (Tregs) remain under-characterized, limiting precise identification of patients who may benefit from immunomodulatory therapies. Tregs are key mediators of immunosuppression within the tumor microenvironment (TME) and are closely associated with resistance to immune checkpoint inhibitors (ICIs). Therefore, defining and characterizing tumors with predominant Treg-mediated immunosuppression is essential for optimizing the use of Treg-targeted and combination immunotherapies.

**Methods:**

We applied an unsupervised multi-omics integration approach across four molecular layers — mRNA, miRNA, DNA methylation, and proteomics —to identify immunologically distinct subtypes of breast cancer. Autoencoder-based dimensionality reduction followed by consensus clustering revealed a subgroup characterized by high Treg infiltration and immunosuppressive signaling, referred to as the Treg-enriched subtype. To evaluate therapeutic strategies, we employed a spatial quantitative systems pharmacology (spQSP) model simulating tumor–immune dynamics and tested Treg-targeted and PD-1 blockade therapies both alone and in combination. *In vivo* efficacy studies were conducted using the EMT6 syngeneic breast tumor model, characterized by an immunosuppressive tumor microenvironment, assessing the antitumor effects of a CCR8-targeted small molecule (IPG7236) as monotherapy or in combination with anti–PD-L1 treatment.

**Results:**

The C2 cluster exhibited elevated Treg-related signatures and a highly immunosuppressive tumor microenvironment. A similar Treg-enriched cluster was also identified in an independent cohort, supporting the robustness and clinical relevance of this immunosuppressive subtype. In-silico simulations performed under a C2-like, immunosuppressive context predicted that combining Treg-targeted therapy with PD-1 blockade would substantially enhance immune activation and tumor control compared with monotherapy. To experimentally validate these predictions, combination treatment of a CCR8 inhibitor (IPG7236) and anti–PD-L1 antibody demonstrated greater tumor growth inhibition than either monotherapy in the EMT6 model, confirming the predicted therapeutic synergy in Treg-enriched, immune-suppressive tumors.

**Conclusion:**

This study identifies Treg-enriched and immunosuppressive breast cancer subtype through integrative multi-omics analysis and demonstrates, through both in-silico and *in-vivo* approaches, the therapeutic potential of combining Treg-targeted and PD-L1 blockade therapies. These findings highlight Treg-mediated immunosuppression as a key determinant of therapeutic responsiveness, providing a biological rationale for patient stratification and guiding the development of personalized combination strategies for clinical translation.

## 1 Introduction

Breast cancer remains a leading cause of cancer-related mortality worldwide, and its pronounced clinical heterogeneity necessitates precise molecular stratification for optimal therapeutic decision-making (1). Historically, treatment selection has relied on immunohistochemistry (IHC)-based classification using estrogen receptor (ER), progesterone receptor (PR), and human epidermal growth factor receptor 2 (HER2) status (2). The introduction of PAM50, a gene expression-based intrinsic subtyping system, advanced molecular classification of breast cancer by stratifying tumors into basal-like, luminal A, luminal B, HER2-enriched, and normal-like subtypes with established prognostic and predictive value (3, 4). While PAM50 provides a more precise prognostic framework, it predominantly reflects tumor-intrinsic biology and inadequately captures the tumor microenvironment (TME), particularly immune determinants that critically influence therapeutic outcomes (5, 6). Beyond intrinsic subtypes, the TME—comprising diverse cellular and molecular networks surrounding tumor cells—plays a decisive role in shaping tumor progression, immune evasion, and drug resistance (7). Despite advances in immunotherapy, response rates in breast cancer remain modest compared with other tumor types, underscoring the need to better define immunosuppressive subpopulations that drive therapeutic resistance (8).

Several recent studies have attempted to redefine breast cancer subtypes based on multi-omics or immune-related molecular features. For instance, Hu et al. classified breast cancer according to the interplay between cancer stem cell characteristics and the immune microenvironment (9), while Yu et al. identified immune subtypes along an activation axis primarily defined by CD8^+^ T-cell–related gene signatures (10). In contrast, our study establishes a novel classification framework based on the immunosuppressive axis, specifically focusing on regulatory T cell (Treg)-mediated suppression within the tumor microenvironment. This approach highlights a distinct biological dimension of immune heterogeneity that has not been captured by previous immune-based classifications.

Among immune components, Tregs are pivotal in maintaining physiological immune tolerance, yet their accumulation within tumors suppresses cytotoxic T-cell function through checkpoint molecule expression and immunosuppressive cytokine secretion (6, 11). Consequently, Treg infiltration can render immunologically “hot” tumors resistant to immune checkpoint inhibitors (ICIs) despite high immune cell density (12). Therefore, characterizing Treg-enriched tumor subtypes can provide new insights into immunotherapy resistance and inform precision treatment strategies (13).

Building on this multi-omics-based classification, we extended our analysis to investigate therapeutic implications within the immunosuppressive tumor microenvironment. Using an existing spatial quantitative systems pharmacology (spQSP) model of breast cancer (14), we explored how modulation of Treg-related parameters could influence anti-tumor immune dynamics and therapeutic responsiveness. Furthermore, we integrated these *in-silico* findings with experimental validation in a syngeneic mouse model exhibiting features of an immunosuppressive tumor phenotype. This integrative approach establishes a biologically and clinically relevant Treg-enriched breast cancer subtype, providing a quantitative rationale for precision immunotherapy and guiding Treg-targeted combination strategies. Together, this study bridges molecular classification and therapeutic modeling, providing a systems-level framework for translating immunosuppressive biology into precision treatment strategies.

## 2 Materials and methods

### 2.1 Multi-omics identification of a Treg-enriched subtype

#### 2.1.1 Data acquisition and preprocessing

In this study, we obtained multi-omics data for breast cancer (BRCA) from The Cancer Genome Atlas (TCGA) via the Genomic Data Commons (GDC) and from The Cancer Proteome Atlas (TCPA), accessed on March 1, 2025. Specifically, mRNA expression (L1), miRNA expression (L2), and DNA methylation (L3) data were retrieved from the GDC Pan-Cancer Atlas dataset (https://gdc.cancer.gov/about-data/publications/pancanatlas), and protein expression data (L4) were obtained from the TCPA portal (https://tcpaportal.org/tcpa/download.html). Only samples containing complete data across all four omics layers and clinical information were included in the analysis. Features with excessive missing values were removed, and remaining gaps imputed using the K-nearest neighbors (KNN) algorithm (k=5). mRNA and miRNA data were min–max normalized to the [0, 1] range. To reduce dimensionality and retain informative features, we selected 2,000 highly variable mRNA genes, 482 miRNAs, 2,000 methylation probes, and 217 proteins. These features were integrated into a multi-omics dataset for dimensionality reduction and downstream analysis. Since all multi-omics data were obtained from the TCGA Pan-Cancer Atlas, which provides harmonized and batch-adjusted molecular profiles (e.g., EB++ normalization for mRNA), major within-platform batch effects were already minimized. Each omics layer was independently normalized to account for platform-specific scales (min–max normalization for mRNA and miRNA; native beta values for methylation; pre-normalized TCPA protein levels). The autoencoder was trained on this harmonized and appropriately scaled dataset, enabling the learned low-dimensional representation to capture underlying biological structure rather than platform-driven technical artifacts. Accordingly, no additional cross-platform batch correction methods (e.g., ComBat) were applied.

#### 2.1.2 Multi-omics data integration and clustering

Dimensionality reduction was performed using an autoencoder (AE) to manage the high dimensionality of the integrated multi-omics dataset (12). The data were split into training (90%) and validation (10%) sets using a fixed seed. The final AE architecture consisted of a fully connected encoder–decoder network with a 400-dimensional bottleneck layer. The model was trained using mini-batches (batch size=32) for up to 200 epochs with early stopping (patience=5), ReLU activation (15), mean squared error (MSE) loss, and the Adam optimizer with a learning rate of 0.001 to ensure stable convergence (16). To identify robust molecular subtypes, consensus K-means clustering was performed on the low-dimensional embeddings extracted from the AE bottleneck layer. Clustering stability was evaluated using the Proportion of Ambiguously Clustered (PAC) (17) metric across candidate cluster numbers (K=3 to 10), where lower PAC values indicate more stable clustering. To enhance robustness, consensus clustering was repeated 1, 000 times using 80% random subsampling in each iteration.

#### 2.1.3 Immunologic & molecular characterization

To explore molecular and immunologic distinctions among tumor subgroups, differential gene expression analysis was conducted using the DESeq2 package on raw count data (18). Each cluster was compared against the remaining clusters to identify unique transcriptional signatures. Genes with an adjusted p-value < 0.05 and |log2 fold-change| > 1 were considered significantly differentially expressed. Volcano plots were used to visualize the distribution of upregulated and downregulated genes for each comparison.

To characterize the biological functions of these differentially expressed genes (DEGs), Gene Ontology (GO) (19) and Kyoto Encyclopedia of Genes and Genomes (KEGG) enrichment analyses were performed (20). GO terms were categorized into Biological Process (BP), Cellular Component (CC), and Molecular Function (MF) using the clusterProfiler R package. KEGG pathway enrichment was conducted via WebGestalt (https://www.webgestalt.org/), using Homo sapiens (hsa) as the reference (21), and significant pathways were identified using Over-Representation Analysis (ORA) with FDR < 0.05. The top enriched terms and pathways were visualized using bar plots.

Gene Set Enrichment Analysis (GSEA) was employed to investigate global and immunosuppressive pathway activity (22). Hallmark gene sets from the Molecular Signatures Database (MSigDB) were used to identify significantly enriched biological processes (FDR q < 0.05) (23). Additionally, immune regulatory pathways (hsa04060, hsa04650, hsa04658, hsa04659, hsa04660, hsa04662) and a predefined Treg gene set (FOXP3, CCR8, IL2RA, TGFB1, IL10, CCL1, CCL22, CCL17, CXCL12) were analyzed to evaluate immunosuppressive features. Dot plots were used to visualize normalized enrichment scores (NES) and adjusted significance levels (23).

Immune cell infiltration was estimated using TIMER (http://timer.cistrome.org, accessed on March 1, 2025) (24) which performs immune deconvolution through its built-in reference signatures (25). Expression matrices for each cluster (C1–C3) were uploaded individually, the immune estimation matrix was downloaded, and Treg values were extracted and compared across clusters. Treg proportions were extracted and statistically compared across clusters. Immune, stromal, and ESTIMATE scores were also calculated using xCell. Differences among clusters were assessed using the Kruskal–Wallis test, followed by Dunn’s *post-hoc* and Wilcoxon rank-sum tests where appropriate (26, 27).

To characterize the molecular differences among clusters, somatic mutation and copy number variation (CNV) analyses were performed. Somatic mutations were analyzed using the maftools R package (28), and the most frequently mutated genes were identified. Mutations were categorized into different types, including missense, nonsense, frameshift insertions/deletions, and splice-site mutations, and visualized using oncoplots. For CNV analysis, GISTIC2.0 was used to detect significant amplification and deletion events across clusters (29). The G-score was calculated to quantify the frequency of copy number alterations, and genes with recurrent CNV events were identified.

#### 2.1.4 Identification of a Treg-enriched immune-suppressive subtype

To identify the Treg-enriched cluster, we integrated results from GO/KEGG enrichment, GSEA, immune deconvolution, and stromal/immune scoring, selecting the cluster showing the strongest Treg-associated features across all analyses (**Table 1**). This comprehensive framework enabled objective selection of the most immunosuppressive subtype, which subsequently served as the foundation for constructing a simulation environment mimicking its characteristics. This allows evaluation of Treg-targeted therapeutic strategies.

**Table 1:** Selection criteria for identifying the most Treg-enriched cluster.

Analysis type	Method	Selection criteria for treg-enriched cluster
GO Enrichment	Functional enrichment of Treg-associated GO terms (e.g., “regulation of T cell activation”, “positive regulation of cytokine production”)	Cluster with the highest number of enriched Treg-related terms and lowest p.adjust values is selected
KEGG Pathway Analysis	Identification of Treg-related pathways (e.g., IL2-STAT5 signaling, FOXP3 signaling) using enrichment analysis	Cluster with the strongest pathway enrichment (highest NES score) is selected
GSEA	Gene Set Enrichment Analysis (GSEA) of Treg-specific gene signatures	Cluster with the highest NES score for Treg gene sets is selected
Immune Infiltration Estimation	Quantification of Treg proportions using CIBERSORT, xCell, quanTIseq	Cluster with statistically higher Treg proportions (Kruskal-Wallis test & Wilcoxon rank sum test, p < 0.001) is selected
Immune & Stromal Scores	Calculation of Immune Score and Stromal Score using ESTIMATE algorithm	Cluster with the highest immune and stromal scores is selected

Clusters were evaluated using GO/KEGG enrichment, GSEA, immune infiltration, and stromal/immune scores. The cluster with the highest Treg-related enrichment and strongest pathway activation was selected.

#### 2.1.5 External validation of the Treg-enriched subtype

For external validation, we applied the same autoencoder-based dimensionality reduction and consensus K-means clustering pipeline (K=3) to the GSE96058 dataset (30), obtained from the NCBI GEO repository (31), an independent RNA-seq cohort of 3, 069 breast cancer patients. Although only the transcriptomic modality was available, we selected a comparable number of highly variable genes to match the input dimensionality used in the discovery analysis, thereby ensuring algorithmic consistency across cohorts. Treg signature scores were calculated from nine predefined genes representing core Treg identity markers (FOXP3, IL2RA, CCR8) (32), immunosuppressive cytokines (TGFB1, IL10) (33, 34), and Treg recruitment chemokines (CCL1, CCL17, CCL22, CXCL12) (35), selected based on their established roles in Treg biology and tumor microenvironment. Pathway-level enrichment was evaluated using GSEA with immunologic gene sets from the MSigDB C7 collection (23, 36). To assess the reproducibility of molecular characteristics, PAM50 classification was additionally applied.

### 2.2 In-silico simulation of Treg-targeting therapy

To evaluate the therapeutic implications of the omics-defined Treg-enriched subtype prior to *in-vivo* validation, we performed in-silico simulations to evaluate the therapeutic potential of Treg-targeted immunotherapy and its combination with PD-1 blockade in a highly immunosuppressive TME. A previously published spatial quantitative systems pharmacology (spQSP) model for triple-negative breast cancer (TNBC) was employed (37). This hybrid model integrates systemic pharmacokinetic and signaling components with spatially resolved agent-based modeling, allowing for mechanistic simulation of immune–tumor interactions, cytokine dynamics, and treatment effects (14). The model was selected for its ability to reproduce key immune regulatory mechanisms and responses to checkpoint blockade within solid tumors.

To generate a Treg enriched and immunosuppressive baseline, we artificially elevated the values of parameters representing Treg abundance across compartments, including the tumor, lymph node, and circulation. In this configuration, we manually augmented variables corresponding to the Treg population (e.g., V_T_T0, V_LN_T0, V_C_T0) to simulate the initial and systemic dominance of Tregs, thereby reproducing a suppressive immune environment suitable for therapeutic testing.

Therapeutic interventions were modeled by directly adjusting four parameters mechanistically linked to Treg activity. These modifications represent the simulated effects of a hypothetical Treg-targeted therapy that modulates Treg suppression, proliferation, and depletion. The adjusted parameters and their therapeutic implications are summarized in **Table 2**. PD-1 blockade (nivolumab) was modeled by activating nivolumab-specific switches (*nivoOn = 1*), as implemented in the original framework.

**Table 2:** Summary of adjusted and output variables in the QSP simulation.

Category	Parameter	Description	Unit	Default value
Adjusted Variables	q_T0_T_in	Treg influx rate into the tumor compartment	1/(cm³·min)	1.00E-05
	Treg_max	Maximal FoxP3+ T cell density in the tumor	cells/ml	2.00E+06
	Treg-moveProb	Probability of Treg migration	%	0.33
	k_T0_pro	Treg proliferation rate	1/day	1
	k_T0_death	rate of T0 transport into the tumour compartment calculated based on T cell transmigration rate	1/(cm³·min^-^¹)	0.00001
	Nivo_On/Off	Nivolumab treatment status (ICI therapy)	Binary (0=Off, 1=On)	0 (Off)
Output Variables	V_T.C1	Tumor Cell Count over Time	Count	–
	agentCount.Treg.default	Treg cell count in tumor over time	Count	–
	agentCount.CD8.cytotoxic	CD8+ cytotoxic T cell count in tumor over time	Count	–
	death.cancerCell.Stem	Cancer stem cell death count	Count	–

This table presents the key adjustable parameters related to Treg dynamics, along with the outcome variables used to evaluate the effects of Treg-targeted therapy and its potential synergy with nivolumab.

Four simulation scenarios were defined to compare therapeutic contexts: (1) baseline Treg-enriched state, (2) Treg-targeted monotherapy, (3) PD-1 blockade monotherapy, and (4) combination therapy. Simulations were executed over an identical time frame, and outputs were exported as structured files containing molecular-level and cellular-level variables. These data were visualized in R to compare immune activation (e.g., CD8^+^ effector dynamics) and tumor suppression (e.g., cancer cell death) across treatments, enabling evaluation of potential synergistic effects between Treg modulation and PD-1 inhibition.

### 2.3 *In vivo* experimental validation in EMT6

To validate the simulation−derived hypothesis regarding Treg−targeted therapy, we conducted an *in vivo* efficacy study using the EMT6 syngeneic mouse model, which exhibits an immune−excluded tumor microenvironment analogous to the Treg−enriched subtype identified in our multi−omics analysis. Notably, EMT6 tumors are characterized by prominent immunosuppressive components, including Tregs and myeloid−derived suppressor cells (MDSCs), further supporting its suitability for evaluating Treg−targeted strategies (38). For this purpose, we selected IPG7236, a small−molecule CCR8 antagonist currently under preclinical development. IPG7236, a small-molecule CCR8 antagonist developed by *[InnoCare Pharma]*, was selected due to its preclinical efficacy in selectively depleting intratumoral Tregs without affecting peripheral Tregs (39). CCR8 has emerged as a promising target for selectively depleting intratumoral Tregs, and FDA−approved Treg−targeted agents are currently not available in clinical practice (40).

Female BALB/c mice (5 weeks old) were subcutaneously inoculated with EMT6 tumor cells (1 × 10^5^ cells/mouse) into the right flank. Once tumor volumes reached approximately 100 mm³, mice were randomized using a block design based on baseline tumor size (mean ± SD: 96.45 ± 12.93 mm³). Animals were assigned to four treatment groups (n = 5 per group): (1) IgG control (DMSO + 20% HP−β−CD, vehicle containing isotype IgG), (2) IPG7236 (50 mg/kg, p.o., BID), (3) anti−PD−L1 (5 mg/kg, i.p., once weekly × 2), and (4) Combination therapy (IPG7236 + anti−PD−L1, administered concurrently on the same schedule). A separate vehicle-only group was not included, as the IgG control served as the reference arm using an identical formulation and schedule.

Tumor volumes were measured on treatment days 0, 2, 4, 7, 9, 11, and 14 using digital calipers and calculated with the standard formula (L × W² / 2). Body weight was measured at each dosing time point to assess tolerability. The study endpoint was Day 14 or when tumor volume reached ~2, 000 mm³. Statistical analyses were performed using two-way ANOVA (factors: IPG7236, anti-PD-L1, and their interaction). Therapeutic efficacy was assessed by tumor growth inhibition (TGI) on Day 14, normalized AUC (0–14 d), and synergy indices calculated by the Highest Single Agent (HSA) and Bliss independence models. All animal procedures were approved by the Institutional Animal Care and Use Committee (IACUC) and conducted in accordance with institutional ethical guidelines.

## 3 Results

### 3.1 Multi-omics identification of a Treg-enriched subtype

#### 3.1.1 Multi-omics data integration

After applying the preprocessing pipeline, a total of 618 common BRCA samples from TCGA were identified across all four omics datasets. The feature selection and filtering steps reduced the data dimensions while retaining key biological signals. The summary of omics levels, corresponding sample counts, original feature dimensions, and retained dimensions after preprocessing is provided in **Table 3**. The final preprocessed dataset provided a balanced and high-quality data matrix, enabling robust downstream multi-omics integration and clustering.

**Table 3:** Summary of omics data acquisition and preprocessing.

Omics type	Level	Number of samples	Original dimension	Dimension retained
mRNA	Level 1 (L1)	1095	20531	2000
miRNA	Level 2 (L2)	1068	743	482
DNA methylation	Level 3 (L3)	785	396k	2000
Protein expression	Level 4 (L4)	873	224	217
Clinical Data	–	1097	–	–
Total (common samples)	–	618	417k	4699

The table summarizes the number of samples, original feature dimensions, and the retained feature dimensions after preprocessing and feature selection.

#### 3.1.2 Multi-omics data integration and clustering

To integrate the multi-omics datasets, we applied an autoencoder (AE)-based dimensionality reduction, which effectively preserved biologically relevant variations while reducing the risk of overfitting. The selected AE architecture ([4000, 1500, 800, 400]) provided an optimal balance between model complexity and biological interpretability. Training and validation loss curves confirmed convergence and stability (**Supplementary Figure S1**), and a summary of architecture comparisons is provided in **Supplementary Table S1**.

**Figure 1:**
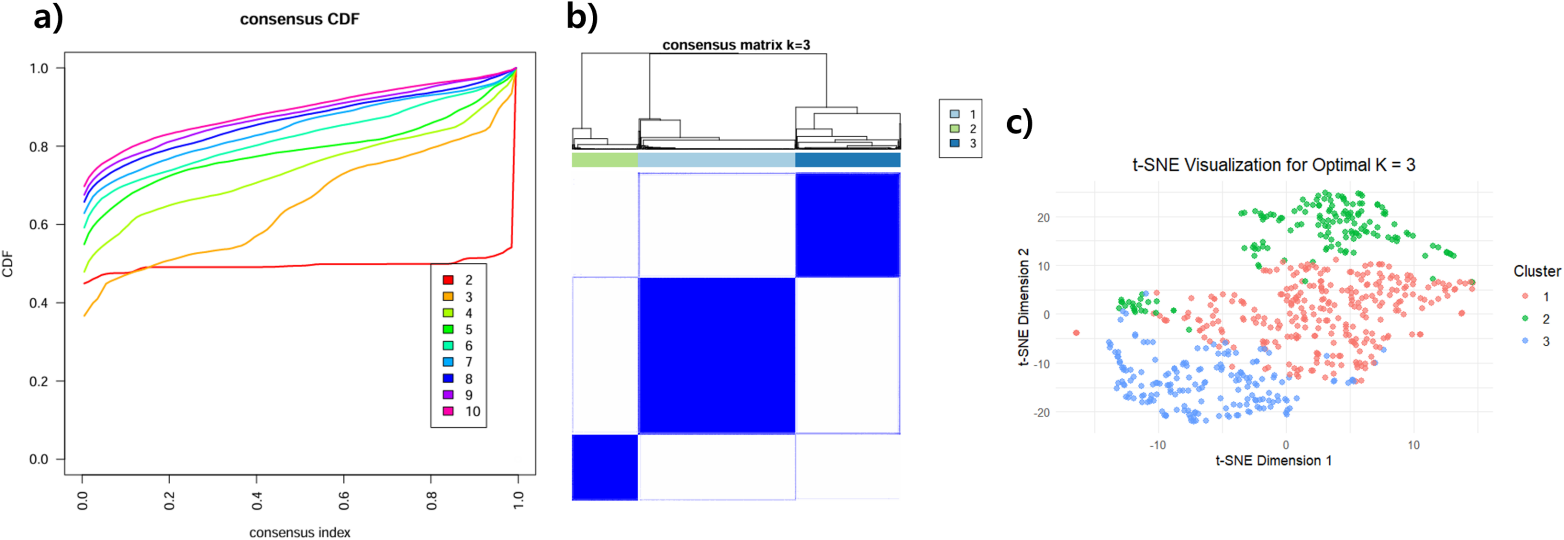
Results of Cluster identification (K=3). The Proportion of Ambiguous Clustering (PAC) metric determined K=3 as the most stable clustering solution, with an optimal PAC value of 0.0417. **(A)** The consensus cumulative distribution function (CDF) curve demonstrates the stability of K=3. **(B)** The consensus clustering matrix shows clear separation among the three clusters. **(C)** t-SNE visualization further supports the distinct partitioning of samples into three clusters.

Subsequent consensus K-means clustering based on the AE-derived latent embeddings identified three stable clusters (K=3), as indicated by the lowest PAC value (0.0417) (**Figure 1a, b**). The consensus cumulative distribution function (CDF) curve, consensus clustering matrix, and t-SNE visualization supported the stability and separation of these clusters (**Figure 1c**). Unlike K=2, which primarily reflected well-known molecular classifications (e.g., Luminal vs. Non-Luminal subtypes), K=3 was able to identify subgroups with potential clinical relevance. These three clusters were hereafter referred to as Cluster 1 (C1), Cluster 2 (C2), and Cluster 3 (C3).

#### 3.1.3 Molecular and immunologic characterization of subtypes

**Figure 2:**
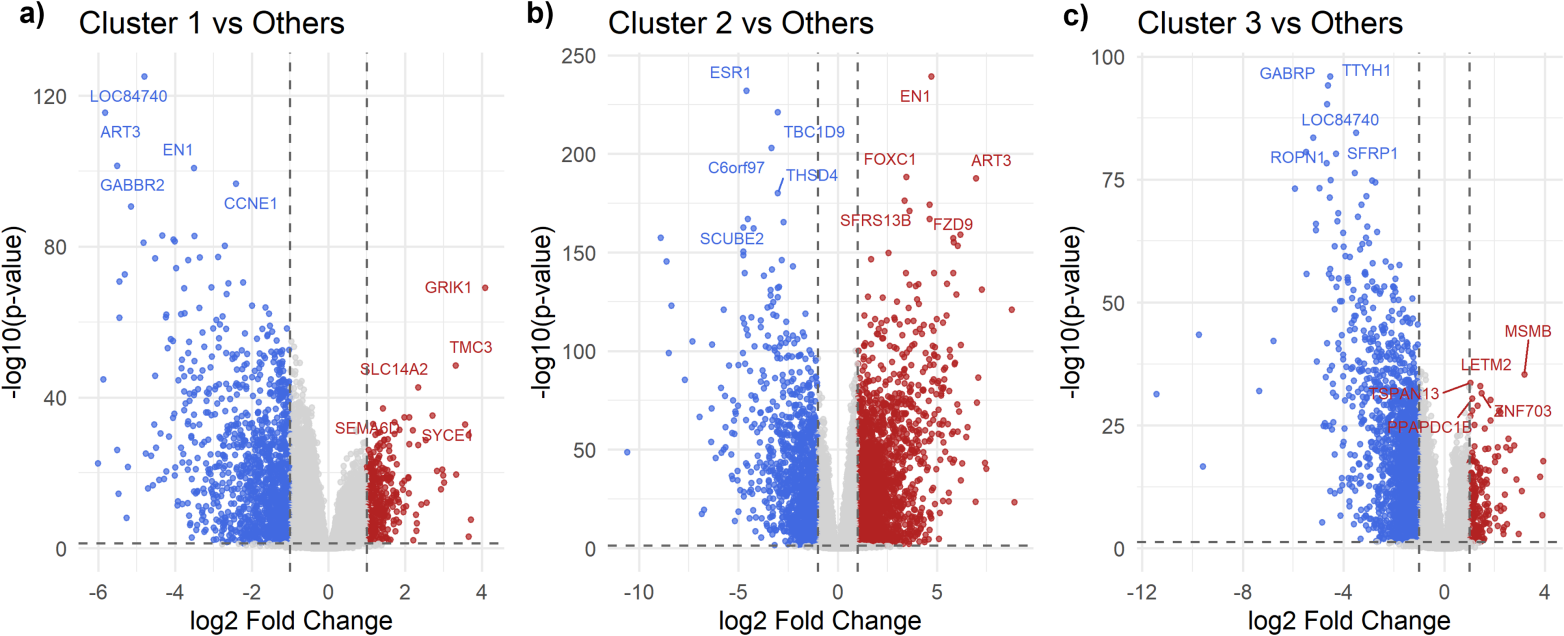
Volcano plots of differentially expressed genes in each cluster: **(A)** C1, **(B)** C2, **(C)** C3. Volcano plots illustrate the DEGs across clusters. The x-axis represents log2 fold change, and the y-axis represents -log10(p-value). Red dots indicate significantly upregulated genes, blue dots indicate significantly downregulated genes, and gray dots represent non-significant genes.

The DEG analysis revealed distinct transcriptional differences among the three clusters. In C1, a total of 344 genes were upregulated, while 1235 genes were downregulated (**Figure 2a**). Notably, GRIK1, SLC14A2, and SYCE1 were among the significantly upregulated genes. C2 exhibited 2353 upregulated and 1402 downregulated genes (**Figure 2b**), with FOXC1, FZD9, and EN1 showing significant upregulation. C3 displayed 216 upregulated and 1738 downregulated genes (**Figure 2c**), with genes such as MSMB, ZNF703, and SPAN13 being highly expressed. Among the three clusters, C2 exhibited a more balanced distribution of upregulated and downregulated genes, while C1 and C3 displayed asymmetric gene expression patterns.

**Figure 3:**
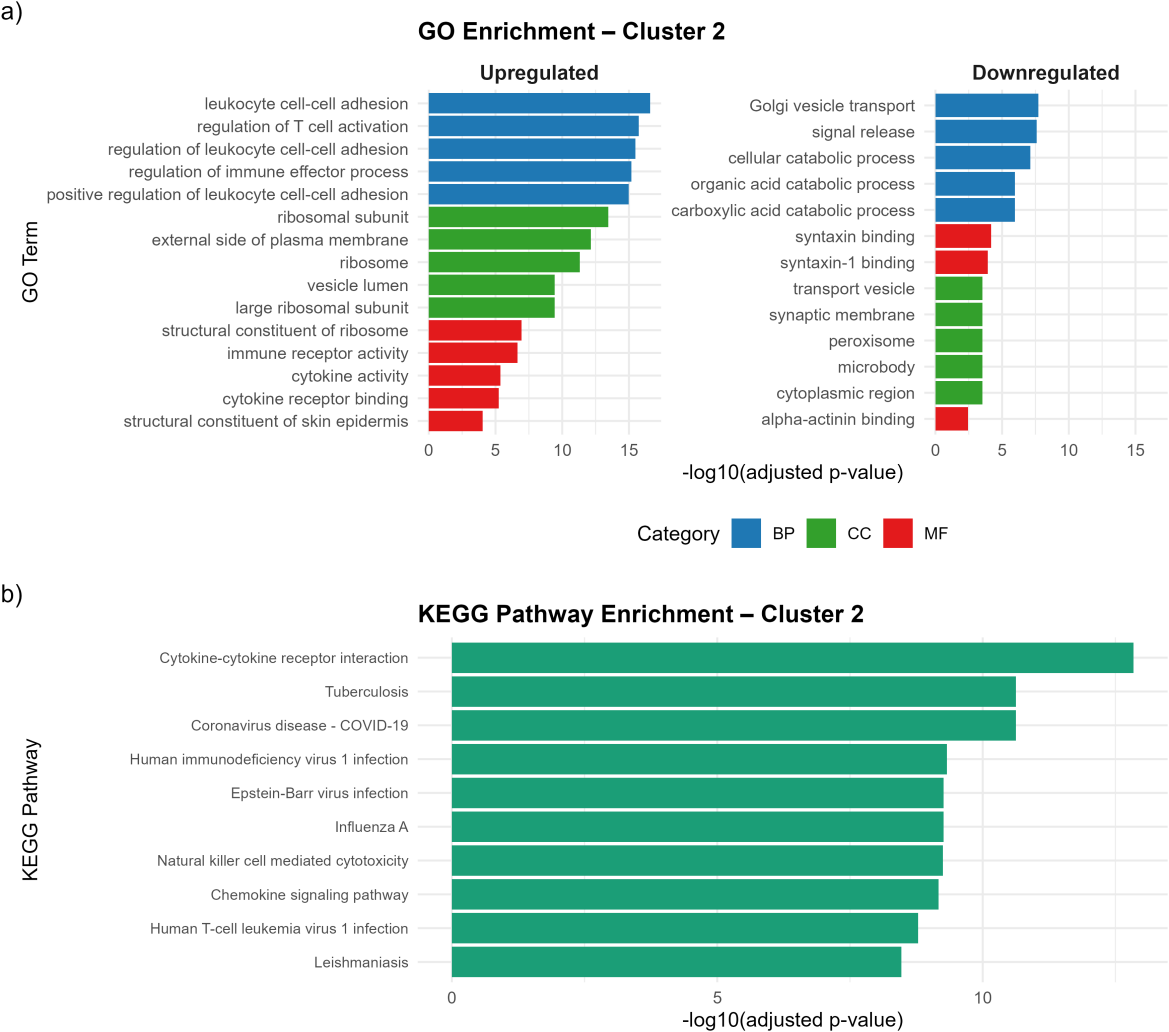
GO and KEGG enrichment analysis of C2. **(a)** GO enrichment of C2, showing top up- and downregulated terms. GO categories are color-coded (BP=blue, CC=green, MF=red). Upregulated GO terms highlight T-cell activation and immune regulation, supporting a Treg-enriched immunosuppressive phenotype. **(b)** KEGG pathway enrichment of C2. Top immune-related pathways (e.g., cytokine-receptor signaling, viral immune response modules) were significantly enriched (FDR < 0.05), indicating active immune signaling despite suppression.

The GO enrichment analysis revealed distinct functional profiles for each cluster. C1 was primarily enriched in biological processes related to extracellular matrix organization and cell adhesion, indicating structural remodeling and stromal involvement within the tumor microenvironment. Additionally, downregulated pathways in C1, including mitochondrial translation and ribosomal biogenesis, suggest reduced metabolic activity, consistent with a stromal-dominant rather than immune-active phenotype (**Supplementary Figure S2**). C2 showed a strong enrichment of immune-related biological processes, with “regulation of cell-cell adhesion”, “regulation of T cell activation” and “positive regulation of cytokine production” among the most significantly upregulated terms (**Figure 3a**). These findings indicate that C2 is characterized by an immune-active microenvironment with enhanced immune cell communication and adhesion, yet this activation is likely dominated by regulatory T-cell (Treg)–mediated immune modulation rather than effective anti-tumor immunity. In contrast, C3 exhibited enrichment in mitochondrial metabolism, proteasome-mediated protein catabolism, and intracellular vesicle transport, suggesting a metabolism-driven phenotype. The downregulation of immune signaling pathways—including cytokine production, immune effector regulation, and chemotaxis—positions C3 as an immune-cold state compared with C2, potentially reflecting metabolic reprogramming rather than immune or stromal signaling (**Supplementary Figure S2**).

KEGG pathway enrichment analysis revealed distinct functional characteristics among the three clusters. C1 demonstrated enrichment in pathways associated with neuroactive ligand–receptor signaling and extracellular matrix–related signaling modules, suggesting receptor-mediated communication and stromal remodeling features consistent with its matrix-dominant phenotype (**Supplementary Figure S3**). C2 exhibited the strongest enrichment in immune-related pathways (**Figure 3b**), including Cytokine–cytokine receptor interaction (hsa04060; Ratio=3.3185, FDR=1.17 × 10^-^¹^4^), Viral protein interaction with cytokine and cytokine receptor (hsa04061), Graft-versus-host disease (hsa05332), and Natural killer cell–mediated cytotoxicity (hsa04650). These findings indicate that C2 is functionally defined by strong cytokine signaling and T-cell–related immune regulation, with activation signatures involving effector pathways (NK cytotoxicity and T-cell stimulation) alongside regulatory cytokine programs (including IL-2/IL-10–associated signaling) known to support Treg expansion and immune suppression. Thus, C2 reflects a dual immune state characterized by high immune activation overlaid with Treg-mediated suppressive regulation — an immune-active but Treg-influenced immunosuppressive phenotype. In contrast, C3 showed predominant enrichment in metabolic and intracellular signaling pathways, including GnRH secretion (hsa04929), proteasome activity, and vesicle transport, consistent with a metabolism-driven phenotype with limited immune engagement (**Supplementary Figure S3**). Overall, while C1 and C3 are characterized by signaling or metabolic programs, C2 is distinctively driven by immune activation coupled with regulatory suppression within the tumor microenvironment. KEGG enrichment results for C1 and C3 are provided in **Supplementary Figure S3**.

**Figure 4:**
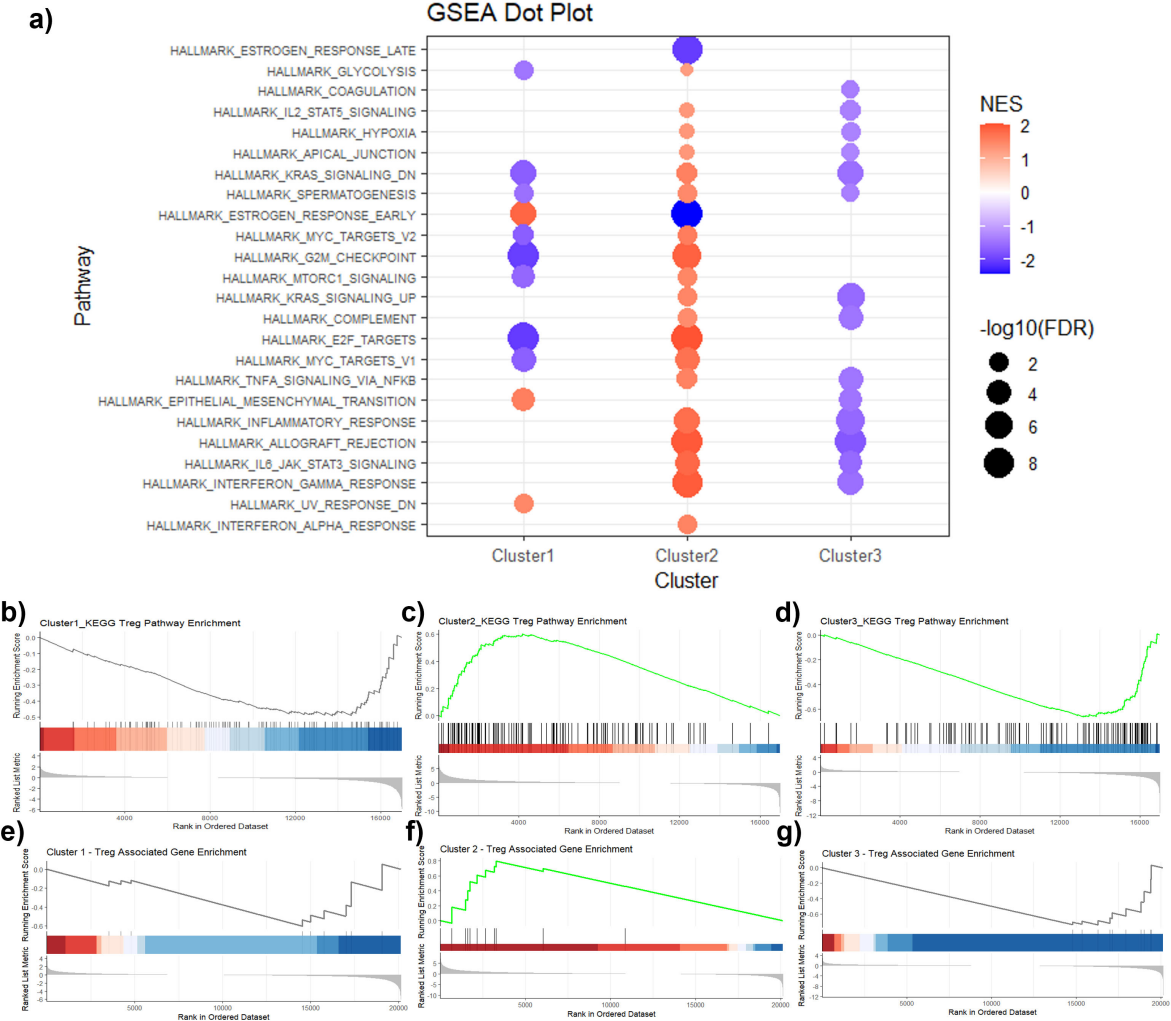
Gene Set Enrichment Analysis (GSEA) across clusters. **(a)** Dot plot of hallmark pathway enrichment across C1–C3. Dot size reflects significance (-log10 FDR), color indicates NES (orange=positive, blue=negative). C2 shows strong enrichment of IL-2/STAT5, IFN-γ, NFκB, and MYC-programs, distinguishing it from C1/C3. Positive NES indicates greater overlap with genes in the corresponding hallmark/KEGG set rather than direct pathway activation. **(b-f)** Representative enrichment curves for Treg-related pathways in each cluster, illustrating dominant activation in C2, with weaker signals in C1/C3.

GSEA was conducted to identify functional differences among the three clusters (21). As shown in **Figure 4a**, Hallmark pathway enrichment analysis revealed distinct pathway signatures, with C1 demonstrating weaker enrichment signals overall but showing relative elevation in epithelial–mesenchymal transition (EMT), extracellular matrix remodeling, and adhesion-related pathways, suggesting a stromal-dominant phenotype with potential tissue remodeling and structural reinforcement. C2 showed significant suppression of effector immune–related pathways and strong enrichment in HALLMARK_MYC_TARGETS_V1, HALLMARK_E2F_TARGETS, and HALLMARK_G2M_CHECKPOINT, indicative of a highly proliferative tumor subtype. Additionally, HALLMARK_TNFA_SIGNALING_VIA_NFKB was enriched, reflecting immune-related signaling activity but in the context of regulatory rather than cytotoxic immune activation. In contrast, C3 showed enrichment of metabolic and hormone-related pathways, alongside reduced interferon and inflammatory signaling, suggesting an immune-cold and metabolism-driven phenotype. To further explore Treg-associated signatures, we conducted targeted GSEA focusing specifically on Treg-related pathways and gene sets. KEGG Treg pathway analysis demonstrated significant enrichment exclusively in C2, indicating a distinctly immunosuppressive environment (**Figure 4d-f**), whereas C1 and C3 showed no meaningful enrichment in these pathways. Additionally, Treg-associated gene set enrichment, involving key regulatory markers (FOXP3, CCR8, IL2RA, TGFB1, IL10), further confirmed this immunosuppressive phenotype, with the highest enrichment observed again in C2 (**Figure 4e-g**), while both C1 and C3 remained largely Treg-inactive. Comparative visualization of immune-related pathway enrichment scores clearly distinguished C2 as possessing a robust Treg-driven immunosuppressive profile rather than a cytotoxic immune-active one, in contrast to the low-Treg profiles observed in C1 and C3. A detailed summary of Treg-related gene enrichment and KEGG Treg pathway enrichment results is provided in **Supplementary Table S2**.

**Figure 5:**
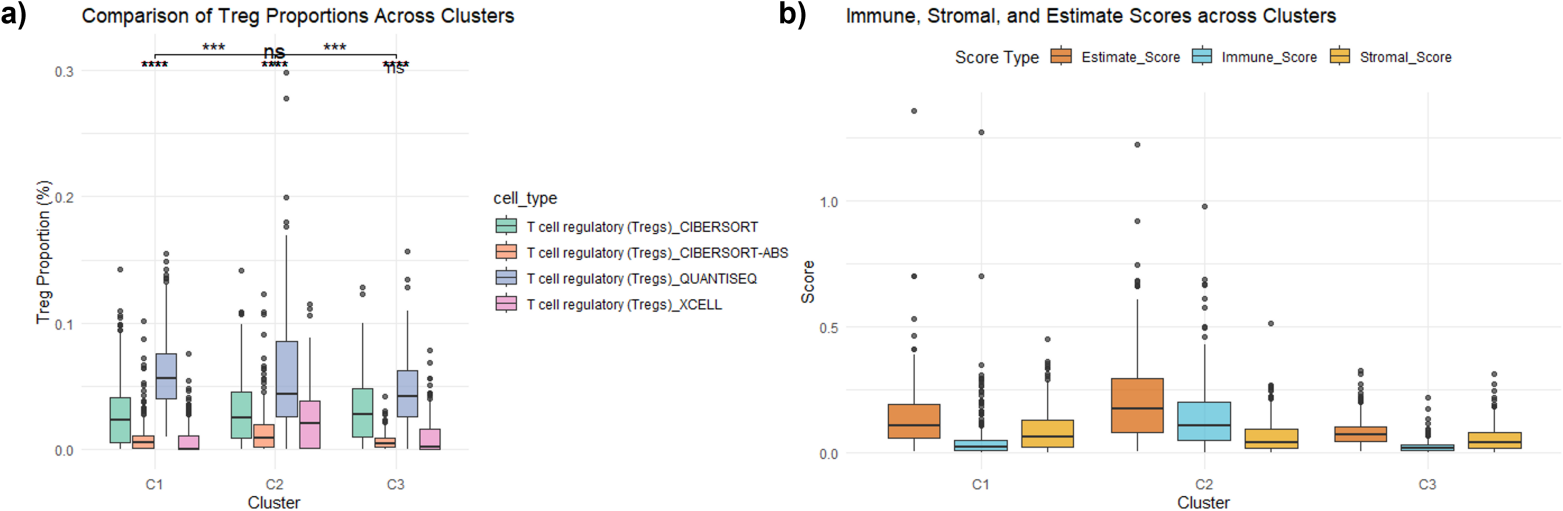
Comparison of Treg proportions and immune scores across clusters. **(A)** Treg proportions across clusters: Boxplots displaying the proportions of regulatory T cells (Tregs) in each cluster, estimated using multiple deconvolution methods (CIBERSORT, CIBERSORT-ABS, quanTIseq, xCell). Statistical significance is indicated (***p < 0.001, **p < 0.01, *p < 0.05, ns=not significant). **(B)** showing the distribution of immune, stromal, and ESTIMATE scores in each cluster, quantifying the tumor microenvironment composition.

Immune infiltration analyses revealed significant differences in Treg proportions across the three clusters (Kruskal-Wallis test, p < 0.001). Subsequent pairwise comparisons using Dunn’s *post-hoc* test demonstrated that C2 had significantly higher Treg proportions compared to C1 (p < 0.00001) and C3 (p < 0.0001), while no significant difference was observed between Clusters 1 and 3 (p=0.82). These findings were further validated by Wilcoxon rank sum tests (both p < 2e-16), confirming C2 as the most Treg-enriched subtype, indicative of a distinctly immunosuppressive tumor microenvironment (**Figure 5a**). Boxplot visualization supported these results, highlighting the immunosuppressive profile unique to C2.

Additional immune scoring analyses, including Immune Score, Stromal Score, and ESTIMATE Score, further delineated the immune-related characteristics of the clusters (**Figure 5b**). C2 exhibited the highest Immune Scores, consistent with its elevated Treg enrichment and immunoregulatory profile. Despite high immune infiltration, the dominance of Treg-associated signatures suggests functional suppression of anti-tumor immunity. In contrast, C1 showed comparatively higher stromal scores and lower immune infiltration, aligning with its ECM- and adhesion-associated biology, whereas C3 demonstrated the lowest immune and stromal scores, consistent with an immune-cold and metabolically driven phenotype. However, Stromal Scores were relatively lower in C2 compared to C1, suggesting a distinct tumor-stroma interaction pattern between these clusters. Collectively, these findings demonstrate C2 as a distinctly immunosuppressive subtype, characterized by enriched Treg populations and corresponding immune signaling activities. Treg proportions significantly differed among the three clusters (Kruskal-Wallis test, p < 0.001).

**Figure 6:**
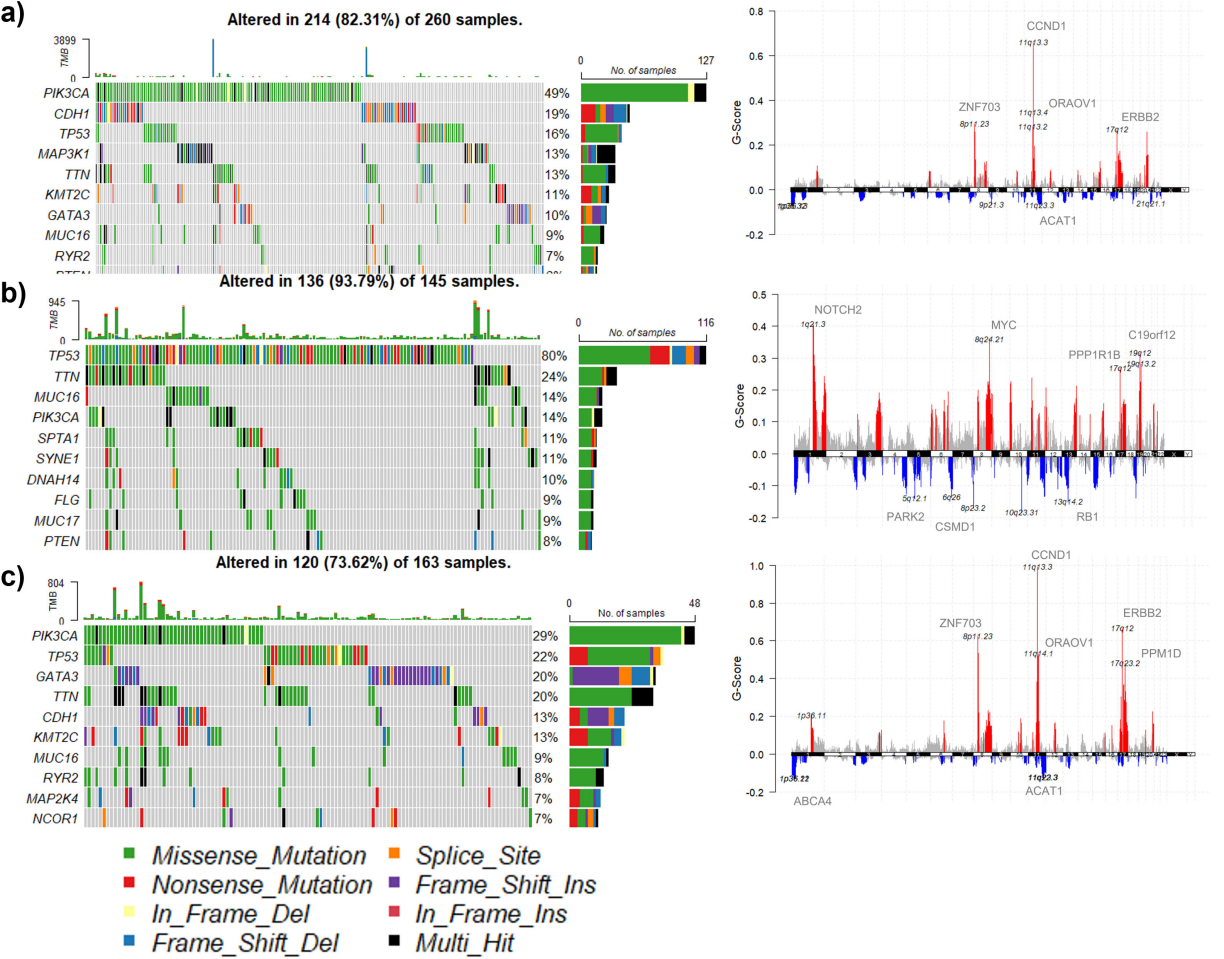
Somatic Alteration Landscape across clusters. **(a–c)** Mutation profiles per cluster. Oncoprints display top altered genes, with alteration types color-coded. C2 displays high TP53/MUC16/PIK3CA mutation frequency, consistent with immune-edited tumor biology. **(d-f)** CNV summary plots. GISTIC analysis highlights amplified oncogenic loci (e.g., MYC, CCND1, ERBB2) and deleted tumor-suppressors, reflecting genomic drivers of immune suppression.

Mutation analysis revealed distinct mutational landscapes across the clusters. C1 was characterized by frequent mutations in PIK3CA, TP53, and MAP3K1 (**Figure 6a**), genes commonly associated with oncogenic signaling. C2 exhibited the highest mutation burden, with TP53, MUC16, and PIK3CA among the most frequently altered genes (**Figure 6b**). C3 showed a relatively lower mutation frequency but retained recurrent mutations in PIK3CA and TP53 (**Figure 6c**). CNV analysis identified distinct patterns of genomic alterations (**Figure 6a-c**). C1 exhibited amplifications in CCND1 and ERBB2, suggesting potential oncogenic activation, whereas deletions were observed in ACAT1. C2 displayed amplifications in MYC and NOTCH2, potentially linked to aggressive tumor phenotypes, along with deletions in RB1 and CSMD1, which are associated with tumor suppressor loss. C3 exhibited amplifications in ZNF703 and ORAOV1, while deletions were detected in ABCA4.

#### 3.1.4 Identification of a Treg-enriched immune-suppressive subtype

To identify the cluster most enriched in Treg-associated features, we performed a multi-dimensional evaluation of immune-related characteristics across clusters (**Table 4**). GO enrichment analysis revealed that C2 had a significant upregulation of Treg-related biological processes, such as “regulation of T cell activation” and “positive regulation of cytokine production, “ which were absent in C1 and C3. KEGG pathway analysis showed that C2 had the strongest enrichment in Treg-specific pathways, as indicated by the highest normalized enrichment score (NES). GSEA further confirmed that C2 exhibited the highest enrichment of Treg signature gene sets. In addition to transcriptional evidence, immune infiltration analysis using CIBERSORT, xCell, and quanTIseq indicated that C2 had a significantly higher proportion of Tregs compared to other clusters. Immune score estimation via the ESTIMATE algorithm also ranked C2 highest among the three groups, further supporting its immune-rich and immunosuppressive tumor microenvironment. Altogether, these findings support the designation of C2 as the most Treg-enriched cluster, distinct from C1 and C3 based on both functional signatures and immune characteristics. These results provide a rationale for selecting C2 as a representative immunosuppressive subtype for subsequent simulation of Treg-targeted therapeutic strategies.

**Table 4:** Comparative analysis of Treg-related features across clusters.

Analysis type	Method	Key metrics	C1	C2	C3
GO Enrichment	Treg-related GO Terms	Presence & p.adjust (-log10)	0 (Low)	Presence detected (High)	0 (Low)
KEGG Pathway	Treg-specific Pathways	NES Score	-0.5 (Low)	0.6 (Strongest)	-0.7 (Low)
GSEA	Treg Signature Gene Set	NES Score	-0.6 (Low)	0.7 (Highest)	-0.7 (Low)
Immune Infiltration	Treg Proportions	% Tregs	0.03–0.04 (Low)	0.05–0.07 (High)	0.03–0.04 (Low)
Immune Score (Estimate)	Immune Score	Score Value	0.30 (Moderate)	0.60 (High)	0.28 (Low)

Table summarizes the key Treg-associated characteristics evaluated across clusters using multiple independent analyses. C2 exhibits the strongest Treg enrichment, as indicated by the highest presence of Treg-related GO terms, strongest pathway activation (KEGG and GSEA), highest Treg proportion, and the highest immune score, distinguishing it from C1 and C3.

Clinically, the Treg-enriched cluster (C2) showed a high proportion of Basal-like/TNBC cases, suggesting that immunosuppressive programs in this subtype may reflect TNBC-associated immune biology rather than Luminal-driven suppression (**Supplementary Figure S4**). This aligns with the well-established association between TNBC and immune-rich but functionally suppressed microenvironments, and provides a rationale for our decision to employ a TNBC-based spQSP model for therapeutic simulation and for validating the synergistic effect of CCR8 blockade with PD-L1 inhibition *in-vivo*.

To strengthen mechanistic interpretation, we further examined whether Treg activity in C2 was linked to effector T-cell exhaustion (**Supplementary Figure S5**). C2 demonstrated the strongest correlation between Treg signatures and exhaustion scores and showed elevated expression of multiple exhaustion markers (PDCD1, CTLA4, LAG3, TIGIT, TOX, ENTPD1) compared with C1 and C3. These results indicate that C2 is not only Treg-rich but functionally immunosuppressed, supporting its classification as an exhaustion-coupled immunoregulatory subtype and providing a clear rationale for Treg-modulating therapeutic strategies.

#### 3.1.5 External validation of the Treg-enriched subtype

**Figure 7:**
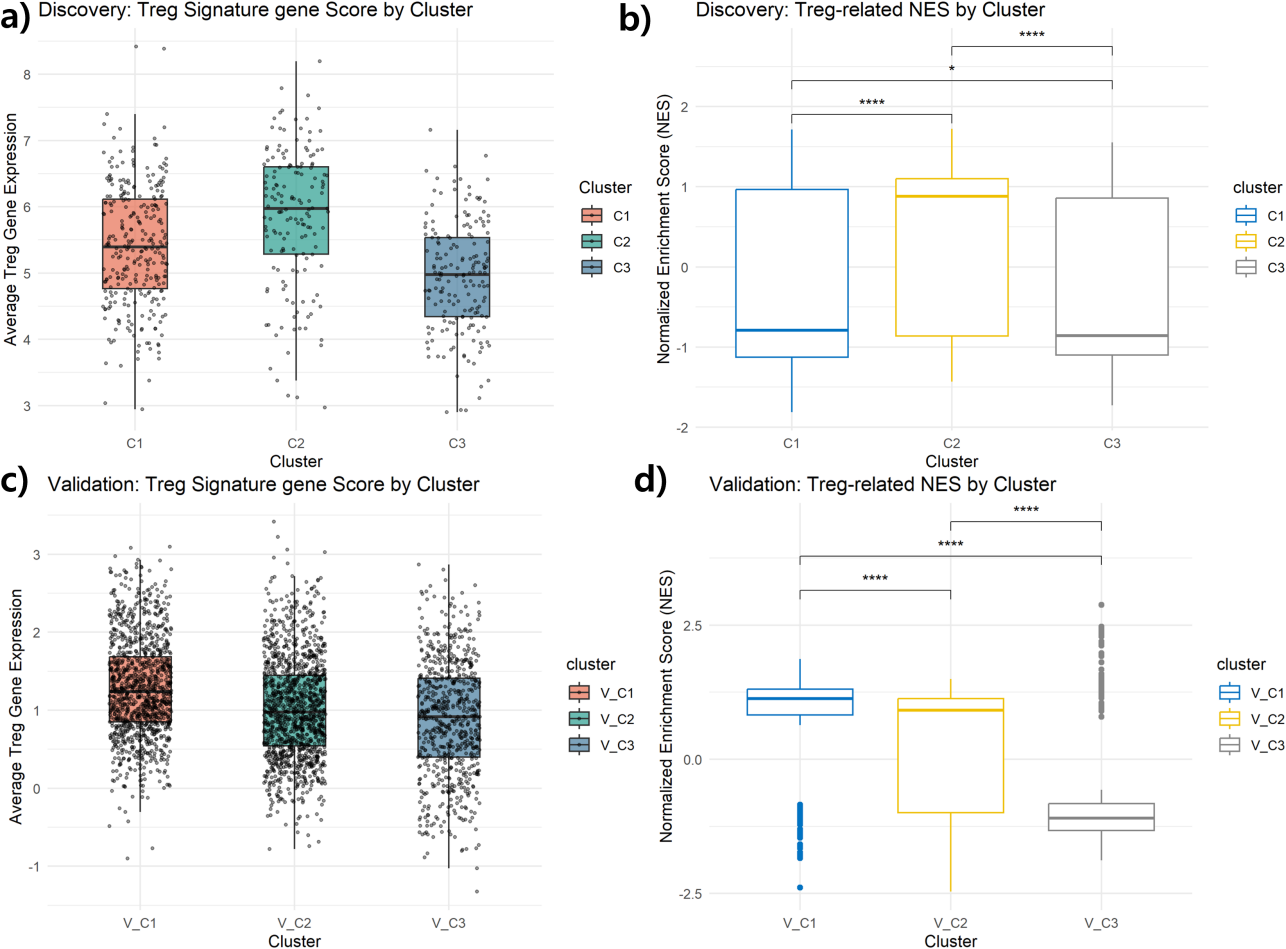
External validation of the Treg-enriched cluster in GSE96058 cohort. **(A)** Average expression of Treg-related genes across clusters V_C1, V_C2, and V_C3. V_C1 shows the highest expression, indicating stronger Treg activity. **(B)** Normalized enrichment score (NES) from GSEA using Treg-related gene sets. V_C1 shows the highest enrichment, while V_C3 shows negative enrichment. ****p < 0.0001.

To externally validate the Treg-enriched breast cancer subtype identified in the TCGA cohort, we applied the same unsupervised clustering (K=3) and Treg-enrichment scoring strategy to the GSE96058 dataset (30), a large independent breast cancer RNA-seq cohort. For direct comparison with the discovery cohort, clusters in the validation cohort were designated as V_C1, V_C2, and V_C3. In the discovery cohort (TCGA), the predefined Treg-associated gene set showed the highest expression in Cluster C2, with significantly elevated Treg signature scores compared to C1 and C3 (p < 0.001, Wilcoxon test; **Figure 7a**). Consistent with this pattern, GSEA using MSigDB C7 Treg-related immunologic gene sets demonstrated that C2 exhibited the strongest positive enrichment, whereas C1 showed negative enrichment (**Figure 7b**). These results confirm that C2 represents the Treg-enriched molecular subtype within the discovery cohort. To quantitatively assess clustering stability and cluster correspondence, we calculated PAC values and silhouette scores for both cohorts (**Supplementary Table S3**). The discovery cohort showed silhouette scores ranging from 0.220 to 0.282, reflecting the inherent molecular heterogeneity of TCGA breast cancer samples. The validation cohort demonstrated higher clustering stability (silhouette scores: 0.325–0.381), with V_C1 achieving the highest score (0.381), supporting its robust identification as a distinct subtype.

As shown in **Figure 7c**, cluster-wise average expression of nine key Treg-related genes revealed that V_C1 exhibited the highest median expression, suggesting stronger Treg-associated gene activity compared to V_C2 and V_C3. Although cluster numbering differed between datasets, the molecular and immune characteristics were concordant with the C2 (Treg-enriched) cluster in TCGA. Supporting this, **Figure 7d** shows the results of GSEA using Treg-related immunologic gene sets, where V_C1 had the highest normalized enrichment score (NES), followed by V_C2, while V_C3 consistently exhibited negative enrichment (NES < 0), indicating reduced Treg-associated immunosuppression. Statistical comparisons between clusters confirmed significant differences (****p < 0.0001). Notably, a subset of V_C1 samples displayed lower Treg-related NES scores (**Figure 7b**). This pattern likely reflects tumor microenvironment heterogeneity, intermediate molecular states near cluster boundaries, and platform-related expression variability. Nevertheless, V_C1 collectively exhibited significantly higher Treg-related NES than V_C2 and V_C3 (p < 0.0001), supporting the robustness of its classification.

Furthermore, PAM50 subtype classification revealed that V_C1 was predominantly composed of Basal-like tumors, mirroring the pattern observed in the discovery cohort, where the Treg-enriched cluster (C2) also exhibited the highest proportion of Basal-like cases, whereas C1 and C3 showed more heterogeneous distributions across Luminal A, Luminal B, and HER2-enriched subtypes (**Supplementary Figure S6**). Collectively, the quantitative clustering stability metrics and convergent molecular signatures demonstrate that V_C1 corresponds to the C2 Treg-enriched subtype, confirming the reproducibility and robustness of this immune-suppressive cluster in an independent external cohort.

### 3.2 In-silico simulation of Treg-targeting therapy

We simulated four therapeutic scenarios to evaluate immune and tumor responses in a highly immunosuppressive TME: (1) Treg-enriched baseline, (2) Treg-targeted monotherapy, (3) PD-1 blockade monotherapy, and (4) Combination therapy.

Each simulation generated two output files: a QSP file for molecular-level pharmacokinetics and signaling, and a STAT file for time-resolved cell-level events such as proliferation, movement, and death. These files were used to visualize temporal changes in key immune and tumor variables across all scenarios (**Supplementary Material 2**). Overall, both monotherapies showed modest effects compared to the Treg-enriched baseline, while combination therapy led to the strongest response—marked by increased CD8+ T cell activity and enhanced cancer cell killing. These trends suggest that Treg modulation and PD-1 blockade may work synergistically to restore antitumor immunity. To better understand these effects, we focused on two key outcomes: CD8+ T cell dynamics (effector, cytotoxic, and suppressed states) and cancer cell death (across stem-like, progenitor, and senescent cells). These variables were selected because they directly reflect immune activation and therapeutic impact on tumor control.

**Figure 8:**
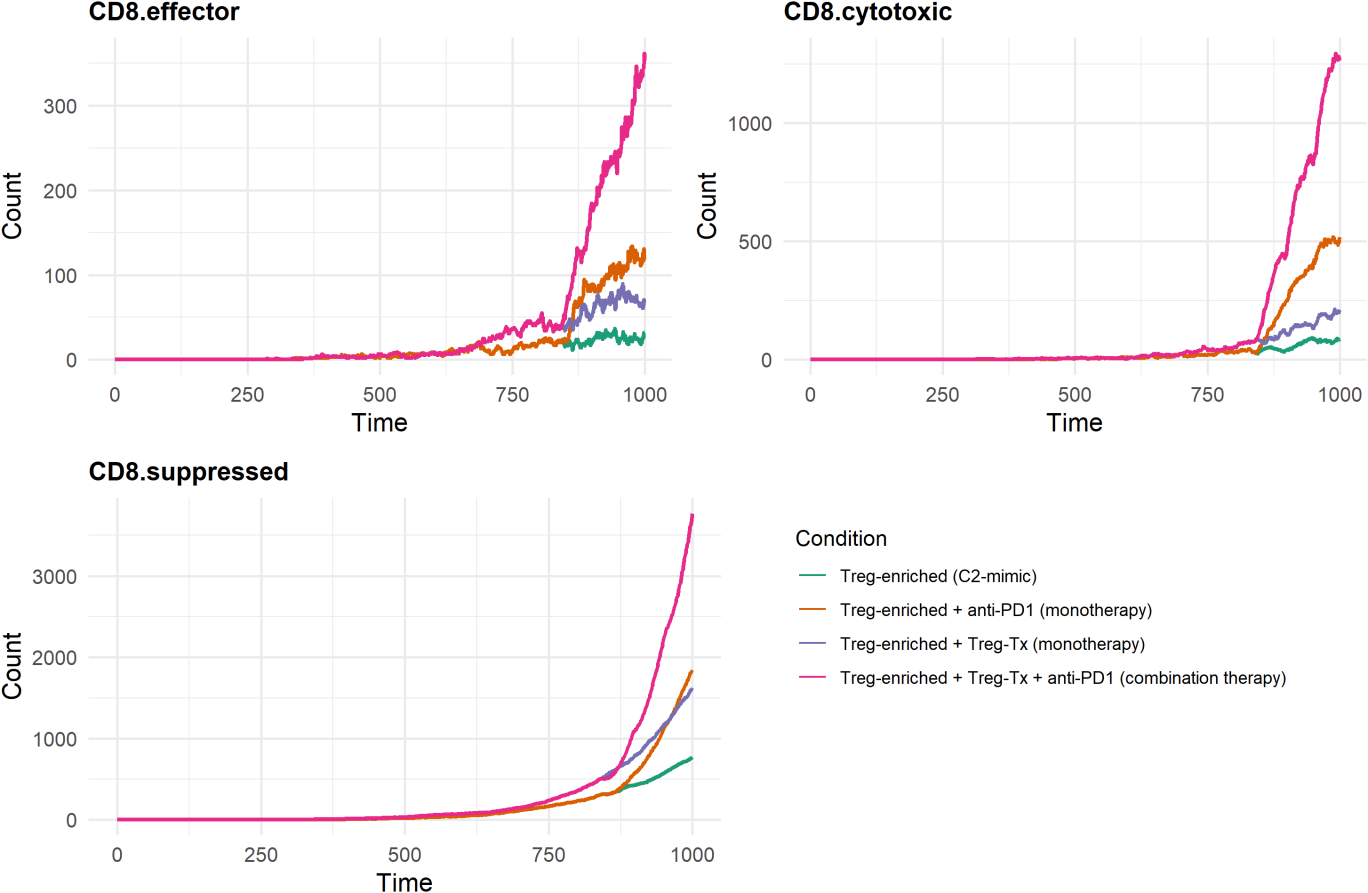
In-silico results of CD8^+^ T-cell dynamics under Treg-targeted and anti-PD-1 therapies. Effector and cytotoxic CD8+ T cell counts were substantially increased by combination therapy, while suppressed CD8+ T cells also expanded, reflecting increased immune activation with residual Treg-mediated inhibition.

Under the Treg-enriched baseline condition—constructed to mimic the immunosuppressive landscape of the C2 cluster—effector, cytotoxic, and suppressed CD8^+^ T cell populations remained consistently low, reflecting a sustained immunosuppressive microenvironment. Treg-targeted monotherapy showed a modest increase across all subsets. PD-1 blockade resulted in a greater increase, particularly in the cytotoxic compartment. The largest increase in CD8^+^ T cell counts was observed in the combination therapy condition, where both effector and cytotoxic subsets expanded notably after simulation time point 750. Suppressed CD8^+^ T cells also increased under this condition. These results suggest that the combination of Treg-targeted therapy and PD-1 blockade is more effective than either monotherapy in increasing CD8^+^ T cell populations across functional states within an immunosuppressive tumor microenvironment (**Figure 8**).

**Figure 9:**
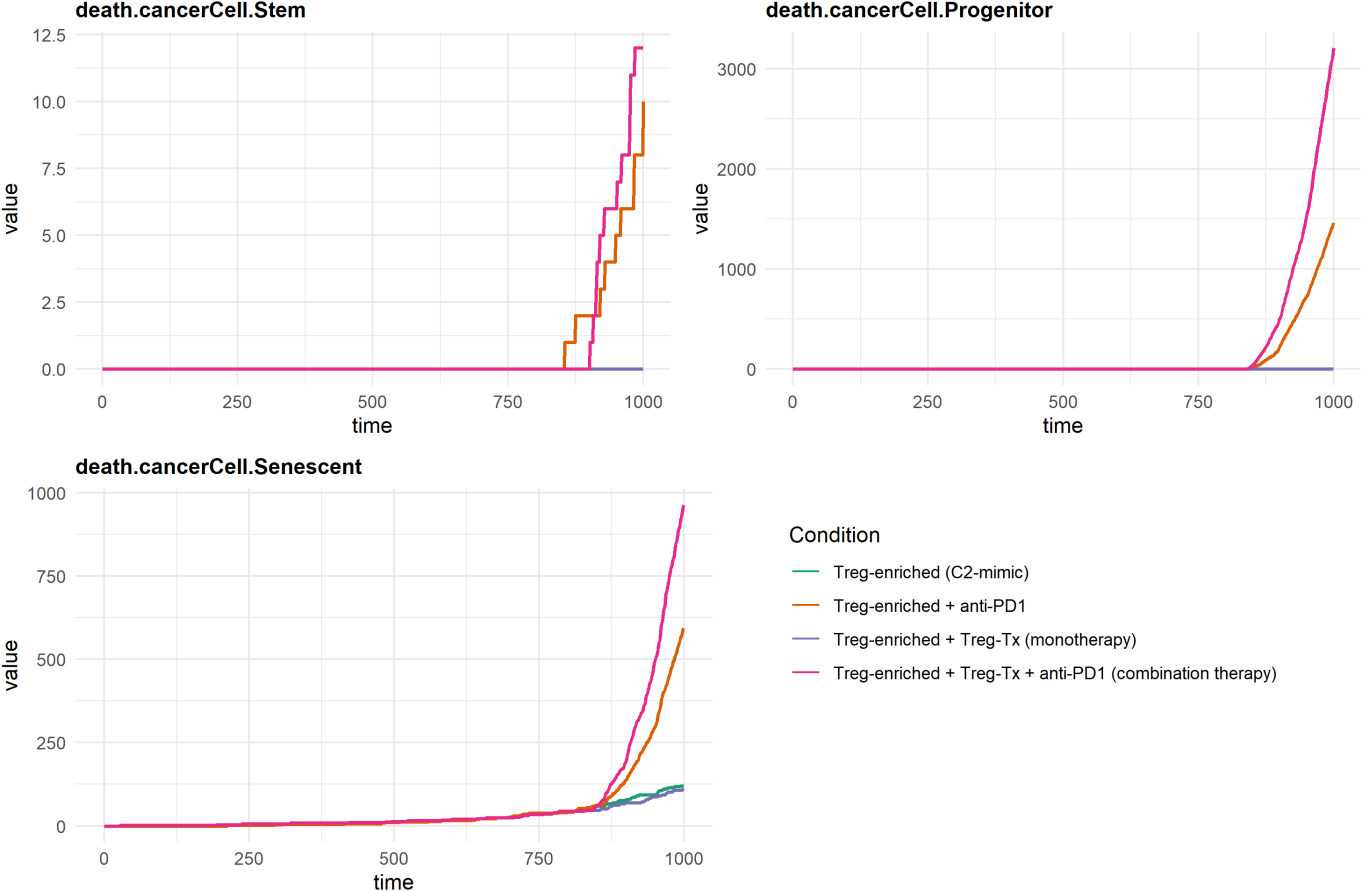
In-silico results of cancer cell death under Treg-targeted and anti-PD-1 therapies. Dynamics of cancer cell death in stem-like, progenitor, and senescent compartments under four conditions. Combination treatment markedly enhanced death across all compartments, particularly in progenitor and senescent cells.

**Figure 9** illustrates the temporal dynamics of cancer cell death across three subtypes—stem-like, progenitor, and senescent—under distinct therapeutic conditions. Similar to the pattern observed in CD8^+^ T cell responses, cancer cell death was minimal under Treg-enriched conditions, suggesting that the tumor microenvironment is highly immunosuppressive. Treg-targeting monotherapy slightly increased cell death, especially in the progenitor and senescent cell populations, suggesting that the monotherapy has limited therapeutic efficacy. PD-1 blockade monotherapy modestly increased cancer cell death, especially in the progenitor and senescent cell populations. In contrast, combination therapy with Treg-targeting and PD-1 blockade dramatically increased cancer cell death in all subtypes. A significant increase was observed in the progenitor population during the later phase of the simulation, accompanied by a moderate increase in senescent cell death and a slight increase in stem-like cell death.

These findings suggest that while PD-1 blockade and Treg-targeting monotherapy alone have limited effects on tumor cell killing, their combined use may potentially enhance anticancer efficacy by synergistically reactivating immune effector functions in a state of reduced immunosuppression.

### 3.3 *In vivo* experimental validation in EMT6

**Figure 10:**
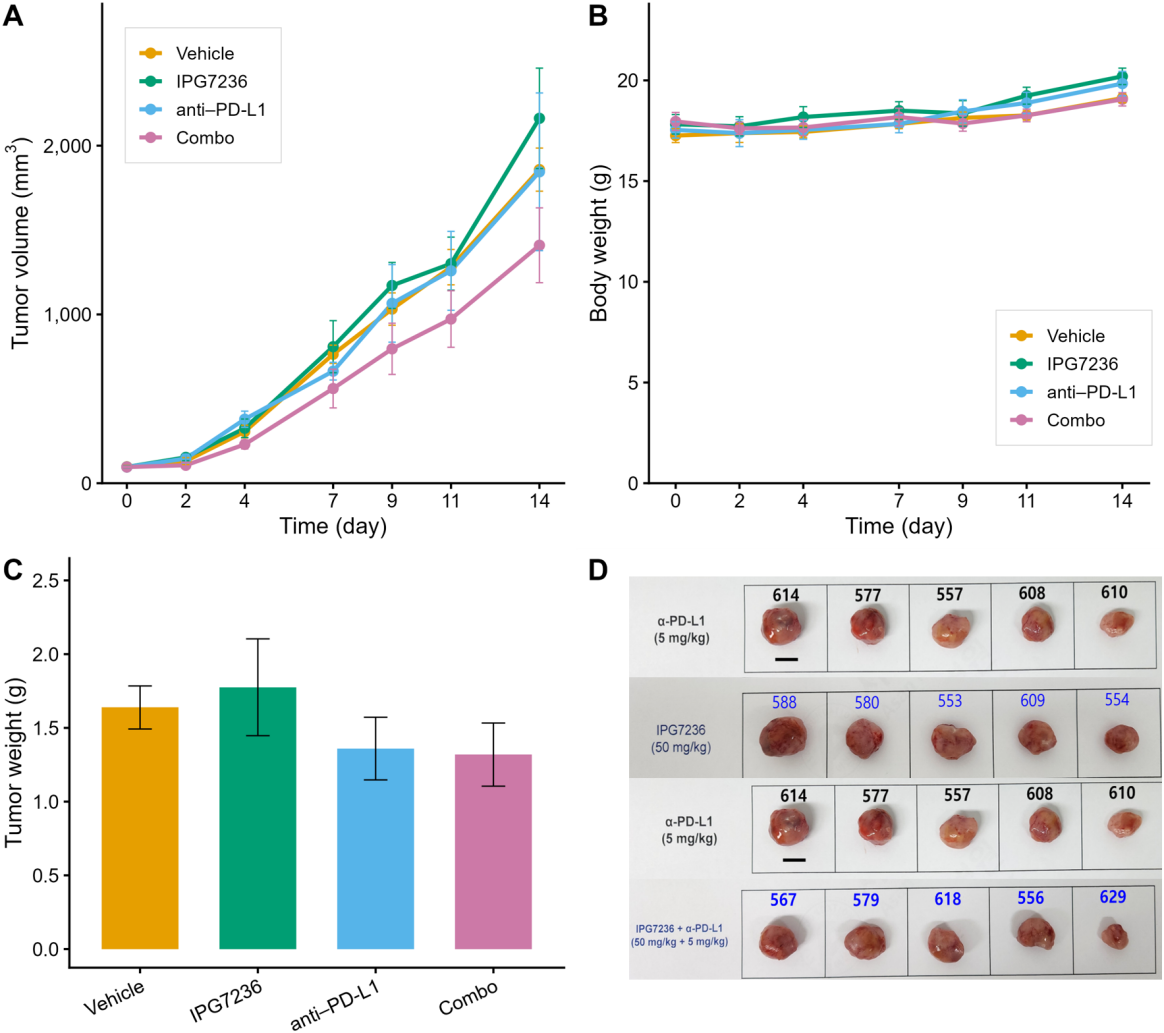
*In vivo* antitumor efficacy of IPG7236 and anti–PD−L1 in the EMT6 syngeneic model. **(A)** Tumor growth curves showing that combination therapy (IPG7236 + anti–PD-L1) produced the greatest tumor growth inhibition compared with monotherapies or vehicle control. **(B)** Body weight changes demonstrating good tolerability across all treatment groups. **(C)** Tumor weights at day 14 confirming the lowest mean tumor burden in the combination group. **(D)** Representative tumor images collected at study endpoint showing visibly smaller tumor size in the combination group.

In the control group, tumor growth continued, reaching a mean tumor volume of approximately 1, 860 mm³ on day 14. IPG7236 or anti-PD-L1 monotherapy slightly delayed tumor progression, but this effect did not persist beyond day 9, and tumor volume ultimately approached the level observed in the control group (~1, 800 mm³). In contrast, the combination of IPG7236 and anti-PD-L1 demonstrated a more pronounced and sustained anti-tumor effect, with a significant difference from the other groups becoming apparent from day 7 onward. At the end of the study, the combination group demonstrated the greatest tumor growth inhibition among all cohorts, with a mean tumor volume reduction of approximately 1, 400 mm³ (**Figure 10A**).

Quantitative evaluation revealed that combination therapy achieved a TGI of +25.4%, whereas IPG7236 and anti–PD–L1 monotherapy resulted in TGIs of –17.3% and +0.7%, respectively. The improvement in efficacy relative to the most active monotherapy (ΔTGI_combo–max(mono)) was +24.7 percentage points. Similarly, area AUC analysis over the 0–14 day period demonstrated a ~24% reduction in tumor burden in the combination group (AUC=0.760), compared with 0.996–1.089 in the monotherapy arms. Synergy analyses further supported these findings. Under the Highest Single Agent (HSA) model, the combination treatment resulted in an additional tumor volume reduction of ~435 mm³. In the Bliss-independent model, the Bliss exceedance coefficient was 0.419, suggesting a synergistic rather than additive interaction between IPG7236 and anti-PD-L1. Tumor weights at day 14 were consistent with tumor volume data, with the combination group exhibiting the lowest mean tumor weight across all arms (**Figure 10C**). Body weights remained stable throughout the study in all groups (**Figure 10B**), and no treatment-related toxicity or >10% weight loss was observed, indicating acceptable tolerability. Representative images of excised tumors at day 14 visually support the superior antitumor effect observed in the combination group (**Figure 10D**).

Although the interaction term in two-way ANOVA did not reach statistical significance (p = 0.243; n=5 per group), the collective evidence from multiple efficacy endpoints supports a cooperative effect of CCR8 blockade in enhancing the response to PD–L1 inhibition in the immunosuppressive EMT6 tumor model. These findings experimentally validate the simulation-derived hypothesis, demonstrating that Treg modulation via CCR8 antagonism can potentiate immune checkpoint blockade in Treg-enriched tumor microenvironments.

## 4 Discussion

Tregs play a critical role in maintaining immunosuppressive conditions within the TME, and high levels of Treg infiltration have been associated with poor clinical outcomes and limited responsiveness to immunotherapy (34, 41). Tregs have been extensively studied for their immunoregulatory roles across a range of pathological conditions, including cancer, autoimmune and allergic diseases, transplant rejection, and graft-versus-host disease (42). In oncology, therapeutic strategies aimed at modulating or depleting Tregs through surface markers or cytokine-mediated pathways are actively under investigation (43). In our previous bioinformatics study, we identified CCR8, IL2RA, TNFRSF4, TNFRSF18, and CD80 as key regulators of Treg function and migration (44). Among these, CCR8 emerged as a particularly promising therapeutic target due to its strong correlation with immune checkpoint molecules and genomic instability markers such as tumor mutational burden (TMB) and microsatellite instability (MSI) (45), suggesting its potential as a mechanistic link between Treg-driven immunosuppression and ICI resistance (46). These findings highlight the potential of targeting Treg-related pathways to enhance the efficacy of cancer immunotherapy, particularly in breast cancer (45, 47).

Despite growing recognition of the immunosuppressive role of Tregs in cancer, current molecular classification systems such as PAM50 do not adequately capture Treg-related features in breast cancer (48). As a result, patients with Treg-dominant tumors may not be accurately identified, limiting opportunities for immunomodulatory interventions (49). To address this gap, we applied an unsupervised multi-omics strategy integrating transcriptomic, epigenomic, and proteomic features to define a novel Treg-enriched breast cancer subtype. This integrated clustering yielded a biologically consistent population characterized by high Treg infiltration, increased immune checkpoint expression, stromal activation, and aggressive clinical behavior. These results highlight the clinical significance of this immunosuppressive subtype as a targetable immunosuppressive subtype not captured by existing classifiers.

To evaluate potential therapeutic responses, we employed a spQSP model originally developed for TNBC (12), which was adapted to mimic a Treg-enriched tumor microenvironment. This model enabled quantitative simulation of dynamic tumor–immune interactions and allowed us to evaluate the longitudinal effects of Treg-targeted and PD-1 blockade therapies, both as monotherapy and in combination. The simulations demonstrated that Treg-modulating monotherapy modestly enhanced CD8^+^ T-cell activity and induced partial tumor cell killing, whereas the combination of Treg-targeted therapy and PD-1 blockade produced a synergistic enhancement of cytotoxic activity and tumor reduction. To validate these predictions experimentally, we conducted *in vivo* efficacy studies using the EMT6 syngeneic breast cancer model exhibiting immunosuppressive features. Consistent with the simulation results, Treg-targeted monotherapy with the CCR8 inhibitor (IPG7236) produced only modest tumor control, reflecting the highly immunosuppressive nature of this model. However, when combined with anti–PD-L1 antibody, the treatment achieved a markedly greater reduction in tumor growth and enhanced immune activation compared with either monotherapy, confirming the predicted therapeutic synergy. These complementary computational and experimental findings collectively indicate that alleviating Treg-mediated suppression can reprogram the immunosuppressive microenvironment, sensitizing otherwise refractory tumors to immune checkpoint blockade. This combinatorial strategy may therefore be particularly effective in Treg-dominant tumors, where checkpoint inhibitors alone provide limited benefit.

Building upon these results, our study outlines an integrated translational pipeline encompassing (1) multi-omics-based subtype identification, (2) mechanistic QSP simulation for therapeutic hypothesis testing, and (3) *in vivo* validation of treatment efficacy. This integrative framework not only establishes the biological rationale for combining Treg-targeted therapy with PD-1/PD-L1 blockade but also provides a foundation for developing predictive biomarkers to guide patient stratification. Together, these findings bridge molecular classification with therapeutic modeling, supporting the clinical translation of Treg-targeted combination immunotherapy in breast cancer.

Importantly, spQSP-based simulation functioned as a pre-experimental decision layer, allowing us to evaluate Treg-high and Treg-low environments before selecting a model for *in-vivo* work. The simulation revealed that combinatorial efficacy (CCR8 inhibition + PD-1/PD-L1 blockade) emerged only under a Treg-rich setting, whereas Treg-low conditions showed no synergistic interaction. This mechanistic insight led us to prioritize EMT6 for experimental validation—not as a constraint, but as a simulation-driven, translationally efficient model choice.

A key strength of this study lies in the multi-omics clustering framework, which enabled the identification of biologically coherent breast cancer subtypes beyond conventional expression-based classifications. By integrating transcriptomic, epigenomic, proteomic, and immune-profiling layers, the resulting clusters reflected not only expression patterns but distinct biological programs, including stromal remodeling, metabolic regulation, and immunosuppressive Treg-mediated signaling. Importantly, the genomic landscape further reinforced the biological relevance of these subtypes. The Treg-enriched C2 subtype demonstrated recurrent amplifications in MYC and NOTCH2 and deletions in RB1 and CSMD1, suggesting that its immune-evasive behavior may be genetically encoded rather than solely microenvironment-driven. MYC-mediated transcriptional programs have previously been linked to PD-L1 upregulation, impaired antigen presentation, and increased recruitment of regulatory T cells, providing a mechanistic explanation for the immunosuppressive and therapy-refractory phenotype observed in C2. In contrast, genomic alterations in C1 and C3 aligned with stromal-dominant and metabolism-driven biology, consistent with their weaker immune activity revealed through pathway analysis. These findings demonstrate that the identified subtypes are not only functionally distinct but also genomically linked, strengthening the rationale for subtype-specific treatment strategies and supporting the clinical relevance of the Treg-dominant subtype. Additionally, C2 displayed key immunogenomic features frequently associated with immune-active TNBC, including basal-like PAM50 distribution, elevated TP53 mutation frequency, IFN-γ/STAT signaling, inflammatory chemokine activity, and dominant Treg infiltration. Although this similarity is not definitive, the convergence of these molecular and immune characteristics suggests a potential biological connection that will require further investigation to clarify.

Several limitations should be acknowledged. First, due to the unsupervised nature of clustering, direct correspondence between cluster labels in the discovery and validation cohorts is not guaranteed. However, the emergence of a distinct cluster with Treg-enriched features in the external dataset—despite label mismatch—supports the reproducibility of this phenotype across independent populations. Second, enrichment of Treg-related activity was inferred from transcriptomic surrogates rather than direct cellular quantification. Nonetheless, pathway-level analyses and consistent immune signatures reinforce the biological validity of the findings. Third, while the QSP model incorporates key cellular and molecular mechanisms of the tumor microenvironment, it does not fully reflect the complex web of immunosuppressive mechanisms that exist *in vivo*. Lately, our in-silico simulation was based on PD-1 blockade, we employed anti–PD-L1 in the *in-vivo* EMT6 model due to its well-established efficacy, availability, and widespread validation in murine immunotherapy studies. Both agents disrupt the PD-1/PD-L1 axis and have shown comparable immunologic effects in preclinical models. Importantly, we note that PD-1 and PD-L1 blockade converge mechanistically on the same signaling axis, reversing T-cell exhaustion through blockade of the PD-1 receptor–ligand interaction. While PD-1 antibodies act directly on T cells and PD-L1 antibodies primarily block tumor- and APC-derived ligand, both generate similar functional outcomes in EMT6-based immunotherapy studies, supporting the translatability of our simulation findings. This conceptual framework provides justification for PD-L1–based *in-vivo* validation despite PD-1–based computational modeling, and highlights that the observed synergy is attributable to checkpoint disruption itself rather than agent-specific differences. Future work may incorporate direct anti–PD-1 testing *in-vivo* to strengthen the link between computational prediction and experimental validation.

To enhance the translational relevance of our findings, future research should expand this integrative framework beyond breast cancer. Both the multi-omics-based subtype definition and the in silico QSP simulation were confined to breast cancer datasets and models — specifically, the TCGA-BRCA cohort, a TNBC-specific spatial QSP model, and the EMT6 murine breast cancer model (14). Although these systems are highly relevant for studying immunosuppressive subtypes such as Treg-enriched TNBC, the applicability of CCR8-targeted interventions across other tumor types remains unexplored. Ultimately, extending this framework to other Treg-enriched malignancies—such as colorectal and gastric cancers—will be essential to validate the generalizability of our findings and refine Treg-targeted strategies for broader clinical translation.

## 5 Conclusion

In this study, we defined a Treg-enriched breast cancer subtype through integrated multi-omics analysis and characterized its immunosuppressive and aggressive characteristics. QSP-based simulations and *in vivo* experiments demonstrated that Treg-targeted therapy, particularly when combined with PD-1/PD-L1 blockade, can enhance anti-tumor immune responses in this subtype. These results support precision immunotherapy strategies personalized for the Treg-enriched tumor microenvironment in breast cancer. Together, these findings establish a translational framework that links molecular stratification with therapeutic design, offering a translational framework for patient stratification and precision immunotherapy in breast cancer.

## Data Availability

All datasets analyzed in this study are publicly available. Multi-omics data including mRNA (L1), miRNA (L2), DNA methylation (L3), mutation profiles, copy number variation (CNV), and clinical annotations were obtained from the GDC Pan-Cancer Atlas (https://gdc.cancer.gov/about-data/publications/pancanatlas). Protein expression data (L4) were retrieved from The Cancer Proteome Atlas (TCPA) portal (https://tcpaportal.org/tcpa/download.html). For external validation, the RNA-seq dataset GSE96058 was downloaded from the Gene Expression Omnibus (GEO) repository (https://www.ncbi.nlm.nih.gov/geo/query/acc.cgi?acc=GSE96058). The QSPIO-TNBC simulation model used for in-silico experiments was based on a previously published framework and cited accordingly. Original data generated during in vivo experiments are available from the corresponding authors upon reasonable request.
