# Supplementary material for: Integrative multi-omics stratification and translational evaluation of Treg-targeted combination immunotherapy in breast cancer
Source: Front Oncol. 2026 Jan 6;15:1731411. doi: 10.3389/fonc.2025.1731411 (PMC12815797; doi:10.3389/fonc.2025.1731411)
Supplement: Supplementary file 1 [file DataSheet1.docx]

Supplementary Material

Integrative Multi-Omics Stratification and Translational Evaluation of Treg-Targeted Combination Immunotherapy in Breast Cancer

Nari Kim ^1†^, Seongwon Na ^1†^, Hyo Jin Lee^2^, Woojin Yi^2^, Ga Won Son^2,5^, Jin Park^2,6^, Jisung Jang^3^, Mihyun Kim^3^, Seong-Yun Jeong^2,6^*, Kyung Won Kim ^1,3,4,6*^

^1^ Biomedical Research Center, Asan Institute for Life Sciences, Asan Medical Center, Seoul 05505, Republic of Korea

^2^ Asan Institute for Life Sciences, Asan Medical Center, Seoul 05505, Republic of Korea

^3^ Trial Informatics Inc., Seoul 05544, Republic of Korea

^4^ Departments of Radiology and Research Institute of Radiology, Asan Medical Center, College of Medicine, University of Ulsan, Olympic-ro 43 Gil 88, Songpa-gu, Seoul, 138-735, Republic of Korea

^5^ Asan Medical Institute of Convergence Science and Technology, Asan Medical Center, University of Ulsan College of Medicine, Seoul 05544, Republic of Korea

^6^ Department of Convergence Medicine, ASAN Medical Center, University of Ulsan College of Medicine, Seoul 05544, Republic of Korea

*** Correspondence:** Seong-Yun Jeong, Kyung Won Kim,
Corresponding Author
[syj@amc.seoul.kr](mailto:syj@amc.seoul.kr), [medimash@gmail.com](mailto:medimash@gmail.com),
^†^These authors have contributed equally to this work

# Supplementary Tables S1

| No. | Nodes in hidden layer | Epochs | Train loss | Val Loss | PAC value (K=3) |
| --- | --- | --- | --- | --- | --- |
| 1 | [4000, 1500, 800, 400] | 84 | 0.025307 | 0.02968 | 0.043167 |
| 2 | [4000, 2000, 1000, 200] | 48 | 0.029343 | 0.031836 | 0.362443 |
| 3 | [3500, 1500, 500, 100] | 44 | 0.029606 | 0.031976 | 0.110541 |
| 4 | [4000, 3000, 1500, 400] | 38 | 0.029474 | 0.032161 | 0.085737 |
| 5 | [3000, 1500, 800, 200] | 51 | 0.029445 | 0.031809 | 0.182856 |
| 6 | [2000, 1000, 400, 100] | 39 | 0.028761 | 0.032321 | 0.46154 |
| 7 | [3500, 1500, 800, 400] | 44 | 0.031328 | 0.032351 | 0.088935 |
| 8 | [2500, 1000, 500, 200] | 66 | 0.025311 | 0.029908 | 0.480501 |
| 9 | [2000, 800, 400, 100] | 71 | 0.025875 | 0.028988 | 0.282623 |
| 10 | [3000, 1200, 600, 200] | 35 | 0.030199 | 0.032088 | 0.295983 |

**Supplementary Table 1. Summarizing the training and validation losses for different autoencoder (AE) architectures.** This table presents a comparison of different autoencoder (AE) architectures used for multi-omics dimensionality reduction. The architectures are characterized by the number of nodes in each hidden layer, the total training epochs, and their corresponding training and validation losses. The Proportion of Ambiguously Clustered (PAC) value for K=3 is also reported as a measure of clustering stability. The AE architecture with the lowest PAC value (No. 1) was selected for downstream clustering analysis, indicating optimal performance in preserving biologically relevant latent features while maintaining stable subgroup identification.

# Supplementary Tables S2

| Cluster | Pathway | NES (Normalized Enrichment Score) | NES (Normalized Enrichment Score) | p-value | FDR q-value | Enrichment Score (ES) |
| --- | --- | --- | --- | --- | --- | --- |
| Cluster 1 | Treg Associated Gene Enrichment | -0.6 | Negative (Low Enrichment) | Significant | Significant | Low |
| Cluster 1 | KEGG Treg Pathway Enrichment | -0.5 | Negative (Low Enrichment) | Significant | Significant | Low |
| Cluster 2 | Treg Associated Gene enrichment | 0.7 | Positive (High Enrichment) | Significant | Significant | High |
| Cluster 2 | KEGG Treg Pathway Enrichment | 0.6 | Positive (High Enrichment) | Significant | Significant | High |
| Cluster 3 | Treg Associated Gene enrichment | -0.7 | Negative (Low Enrichment) | Significant | Significant | Low |
| Cluster 3 | KEGG Treg Pathway Enrichment | -0.7 | Negative (Low Enrichment) | Significant | Significant | Low |

**Supplementary Table 2. Summary of Treg-Associated Gene and KEGG Treg Pathway Enrichment.** The analysis demonstrates significant enrichment of Treg-related signatures, with high enrichment scores and statistical significance. These findings further support the immunosuppressive phenotype of Cluster 2, distinguishing it from other clusters.

# Supplementary Tables S3

| **Cohort** | **Metric** | **K=3 Overall** | **Cluster 1** | **Cluster 2** | **Cluster 3** |
| --- | --- | --- | --- | --- | --- |
| Discovery (TCGA) | PAC | 0.0417 | - | - | - |
| Discovery (TCGA) | Silhouette Score | 0.258 | 0.2820037 | **0.2547933** | 0.2202953 |
| Validation (GSE96058) | PAC | 0.1355 | - | - | - |
| Validation (GSE96058) | Silhouette Score | 0.366 | **0.3808146** | 0.3251621 | 0.3591544 |

**Supplementary Table 3.** PAC, Proportion of Ambiguously Clustered (lower values indicate more stable clustering); Silhouette scores range from -1 to 1, with higher values indicating better cluster separation. C2 and V_C1 represent the Treg-enriched subtypes in discovery and validation cohorts, respectively.

# Supplementary Figures S1


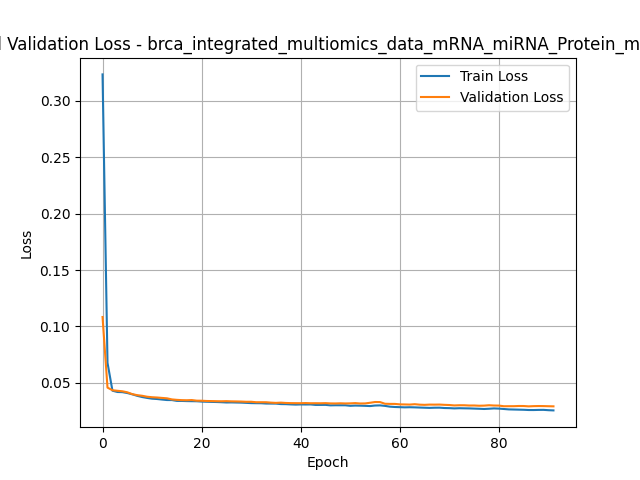


**Supplementary Figure 1. Training and validation losses of autoencoder.** This figure depicts the training and validation loss curves for the autoencoder (AE) model used in multi-omics dimensionality reduction. The loss value decreases steadily over epochs, indicating stable convergence of the model. The minimal gap between training and validation loss suggests that the model effectively captures biologically relevant features while avoiding overfitting.

# Supplementary Figures S2


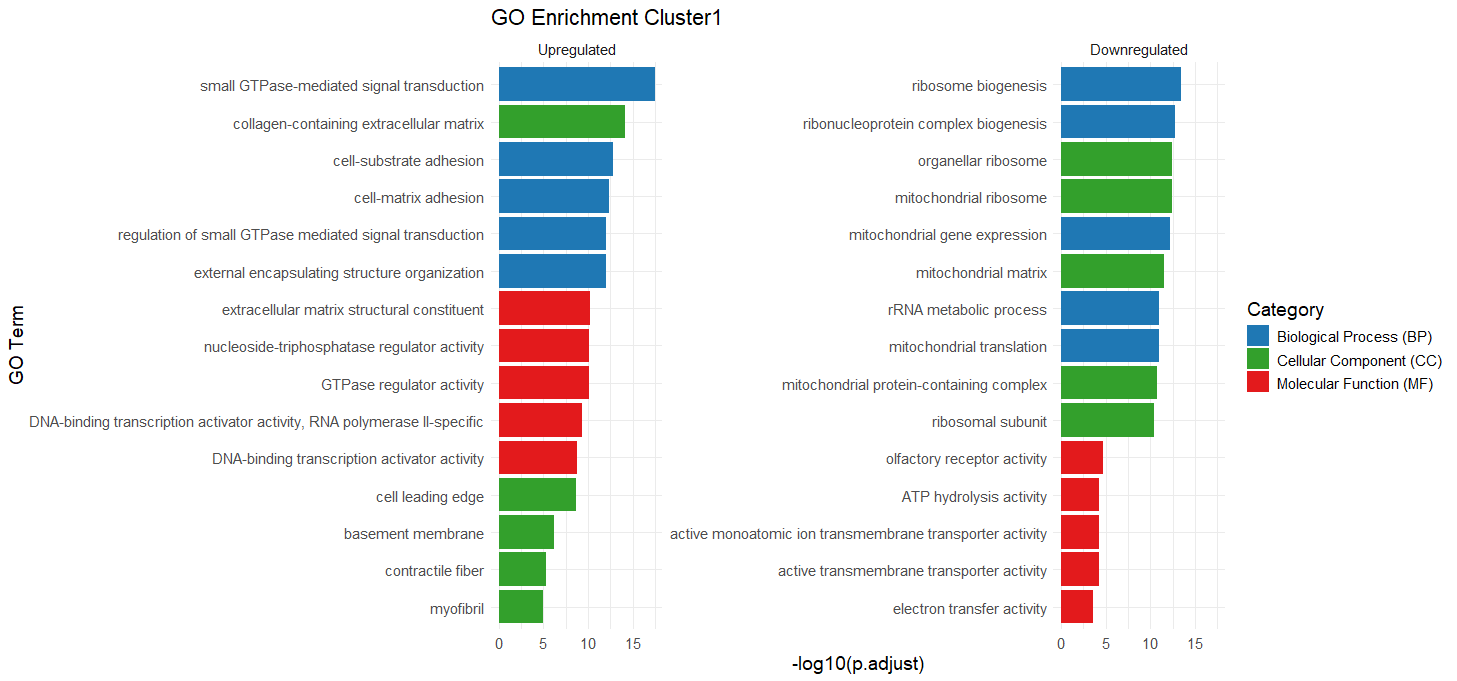


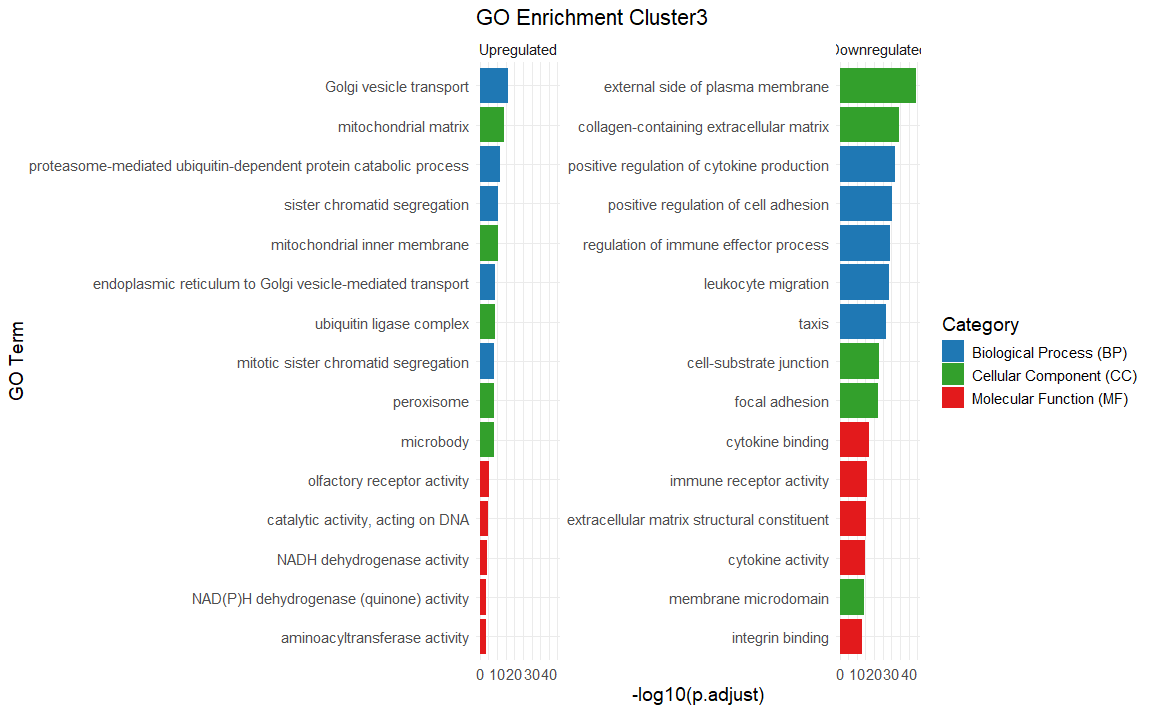


**Supplementary Figure 1. Gene Ontology (GO) Enrichment Analysis of Cluster 1 and Cluster 3.** Cluster 1 exhibited enrichment in extracellular matrix organization and cell adhesion pathways (Up), while Cluster 3 was primarily enriched in metabolic and intracellular transport processes (Down). Downregulated terms in Cluster 3 included immune-related processes, such as cytokine production and immune effector regulation, indicating a less immune-active environment compared to Cluster 2.

# Supplementary Figures S3


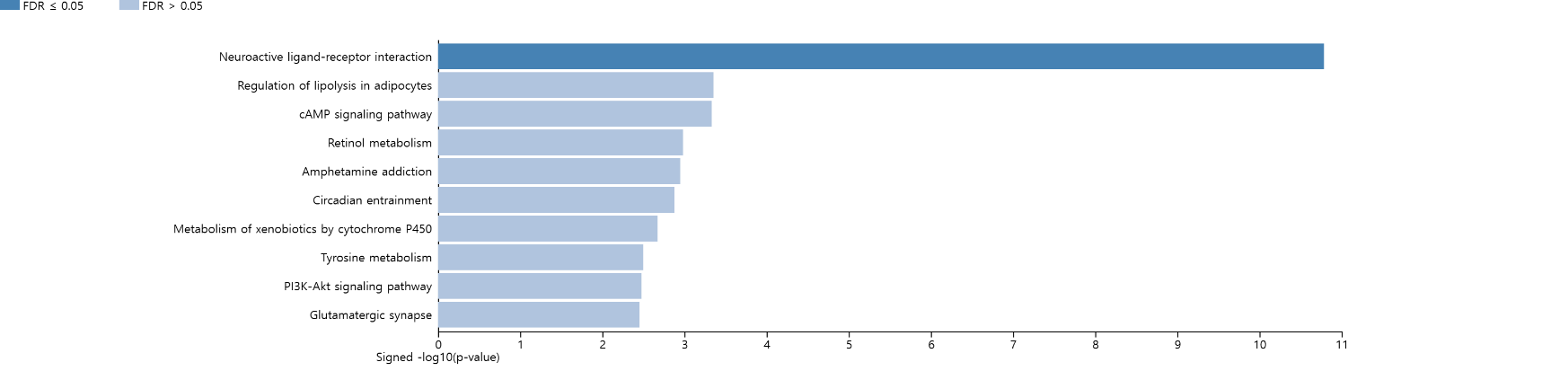

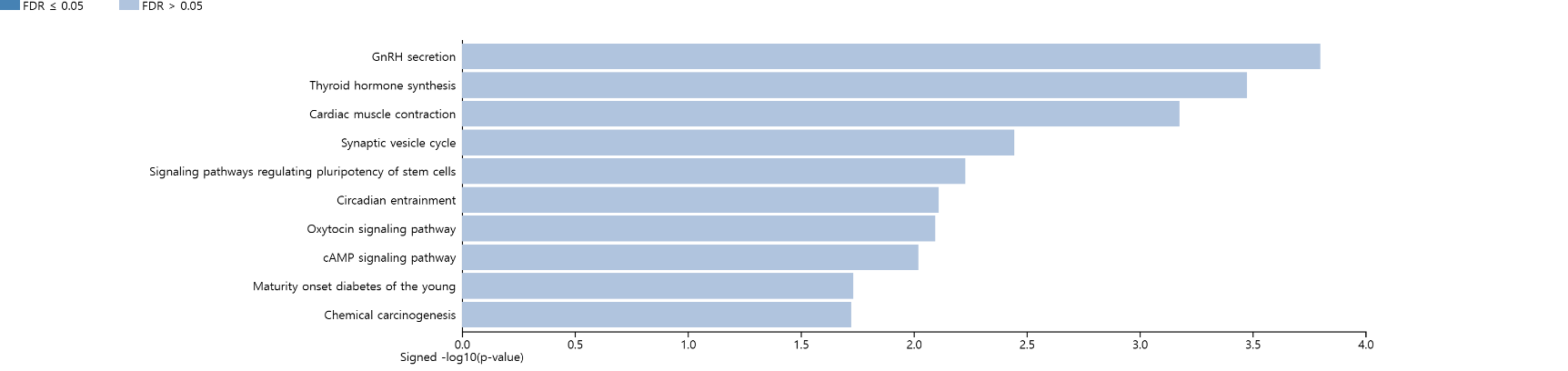


**Supplementary Figure S3. KEGG Pathway Enrichment Analysis of C1 and C3 Clusters.** (Up) KEGG pathway enrichment analysis for Cluster 1, highlighting the top significantly enriched pathways. (Down) KEGG pathway enrichment analysis for Cluster 3, illustrating the key upregulated pathways. Cluster 1 shows strong enrichment in extracellular matrix organization and signaling pathways, while Cluster 3 exhibits enrichment in metabolic and intracellular transport-related pathways.

# Supplementary Figures S4


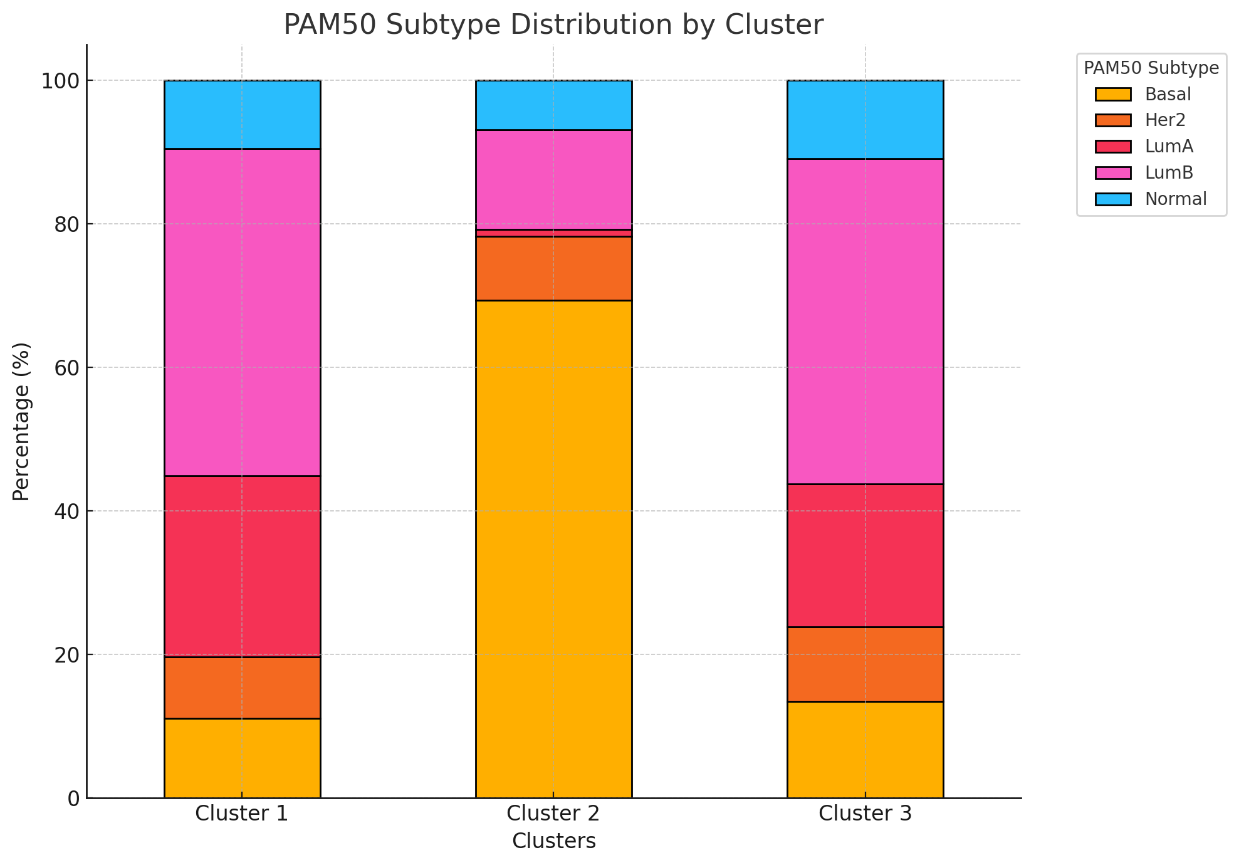


**Supplementary Figure S4. PAM50 subtype distribution across identified clusters**. Stacked bar plots show the proportion of each PAM50 molecular subtype (Basal-like, HER2-enriched, Luminal A, Luminal B, and Normal-like) within each of the three clusters. Cluster 2 (C2), identified as the Treg-enriched subtype, exhibits a high proportion of Basal-like tumors, whereas Clusters 1 and 3 show a more balanced distribution across Luminal and HER2 subtypes.

#
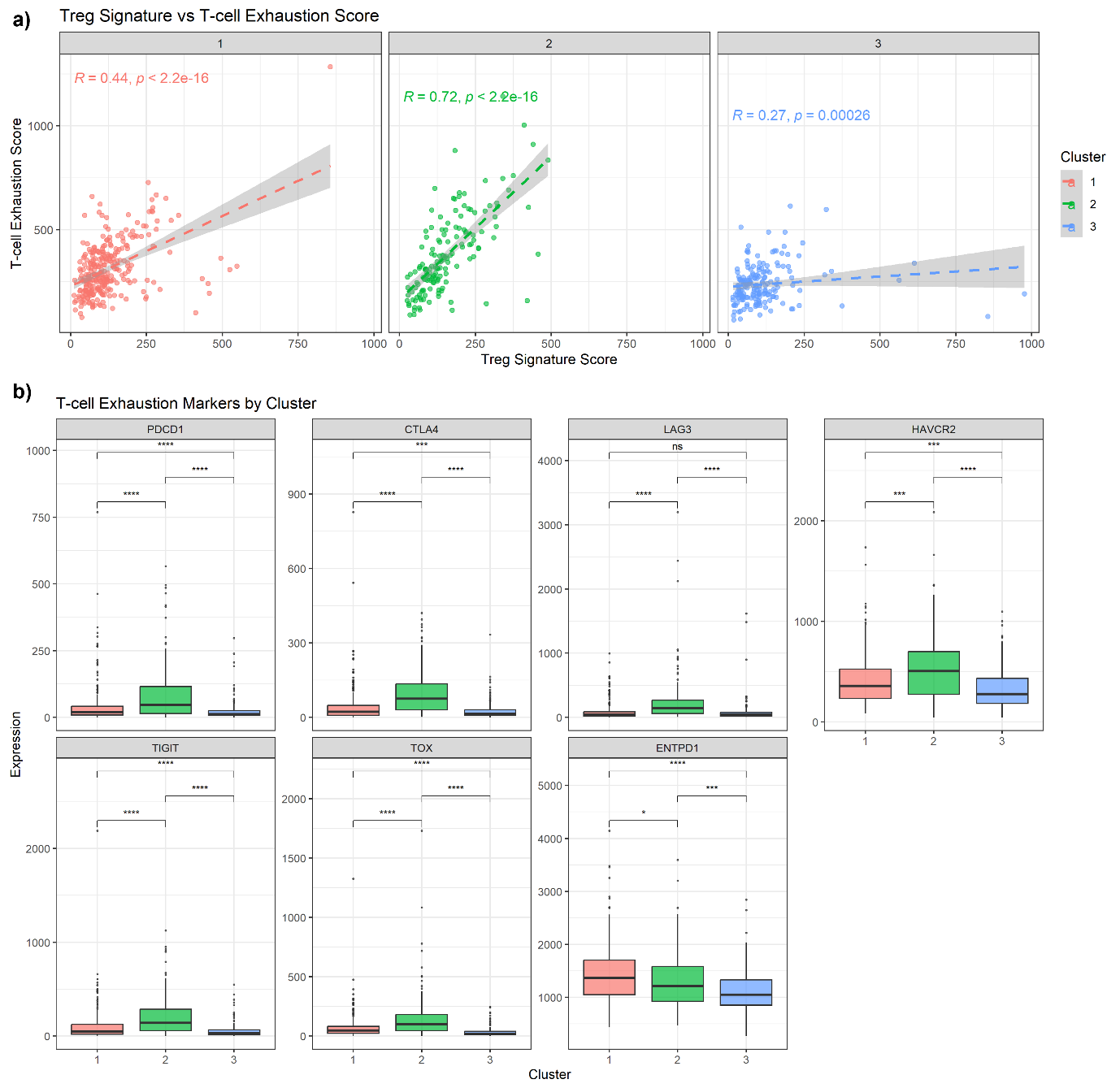
Supplementary Figures S5

**Supplementary Figure S5. Integrated analysis of Treg signaling and T-cell exhaustion across clusters.** **(a)** Correlation between Treg signature score and T-cell exhaustion score in Clusters 1, 2, and 3. Linear regression analysis (colored by cluster) shows a markedly stronger association in Cluster 2 (R = 0.72, p < 2.2e-16) compared to Cluster 1 (R = 0.44, p < 2.2e-16) and Cluster 3 (R = 0.27, p = 0.00026), indicating that Treg-high tumors preferentially co-express exhaustion programs. **(b)** Expression of canonical T-cell exhaustion markers (PDCD1/PD-1, CTLA4, LAG3, HAVCR2/TIM-3, TIGIT, TOX, ENTPD1/CD39) across clusters.

# Supplementary Figures S6


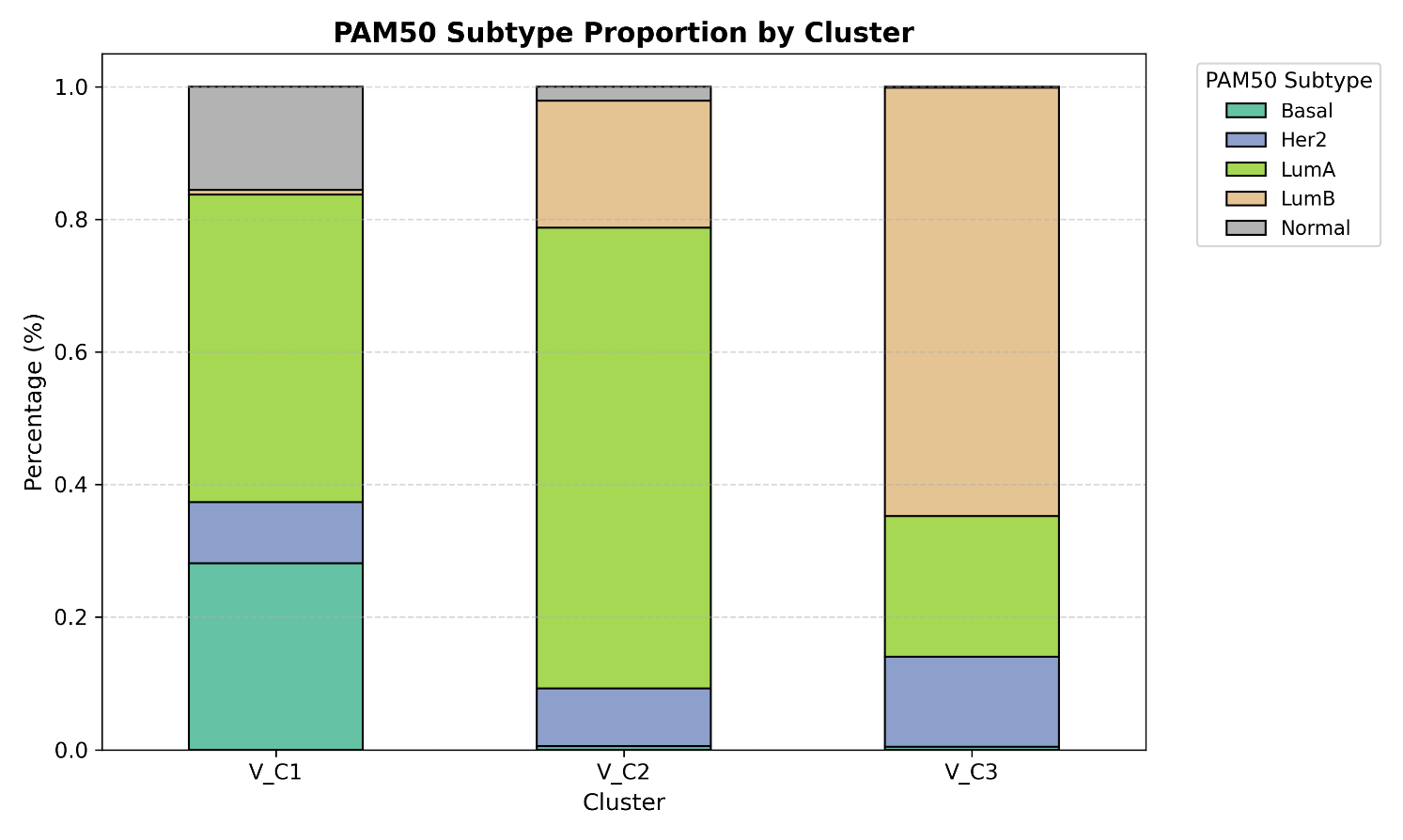


**Supplementary Figure S6. PAM50 subtype distribution across validation clusters.** Stacked bar plots show the distribution of PAM50 subtypes across the three clusters. V_C1, which exhibited Treg-enriched features, included a higher proportion of Basal-like and Normal-like tumors, while V_C2 was dominated by Luminal A, and V_C3 by Luminal B and HER2-enriched subtypes.
