# Supplementary material for: Integrative multi-omics stratification and translational evaluation of Treg-targeted combination immunotherapy in breast cancer
Source: Front Oncol. 2026 Jan 6;15:1731411. doi: 10.3389/fonc.2025.1731411 (PMC12815797; doi:10.3389/fonc.2025.1731411)
Supplement: Supplementary file 2 [file DataSheet2.pdf]

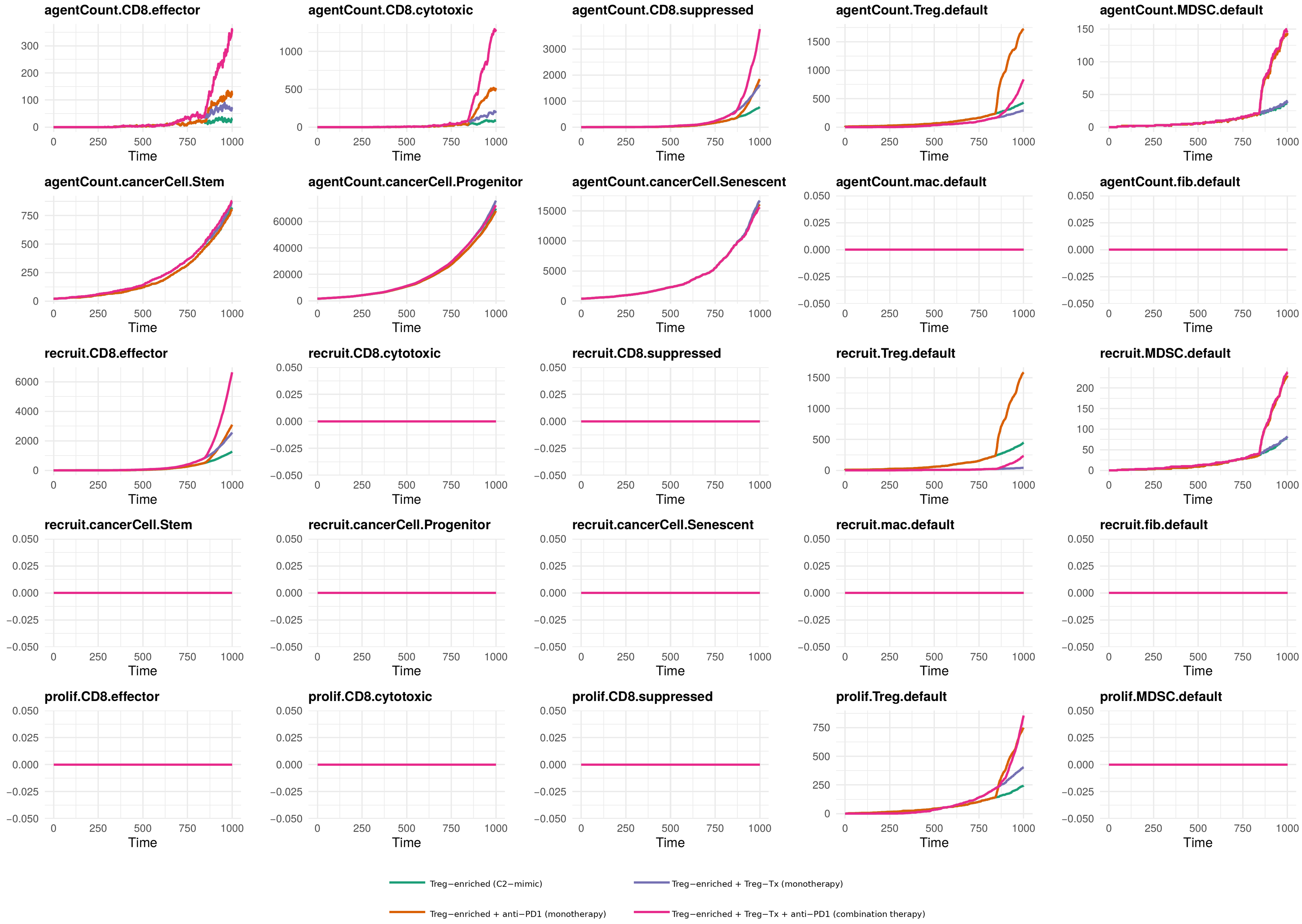
agentCount.CD8.effector
agentCount.CD8.cytotoxic
agentCount.CD8.suppressed
agentCount.Treg.default
agentCount.MDSC.default
agentCount.cancerCell.Stem
agentCount.cancerCell.Progenitor
agentCount.cancerCell.Senescent
agentCount.mac.default
agentCount.fib.default
recruit.CD8.effector
recruit.CD8.cytotoxic
recruit.CD8.suppressed
recruit.Treg.default
recruit.MDSC.default
recruit.cancerCell.Stem
recruit.cancerCell.Progenitor
recruit.cancerCell.Senescent
recruit.mac.default
recruit.fib.default
prolif.CD8.effector
prolif.CD8.cytotoxic
prolif.CD8.suppressed
prolif.Treg.default
prolif.MDSC.default
Time
Treg−enriched (C2−mimic)
Treg−enriched + Treg−Tx (monotherapy)
Treg−enriched + anti−PD1 (monotherapy)
Treg−enriched + Treg−Tx + anti−PD1 (combination therapy)

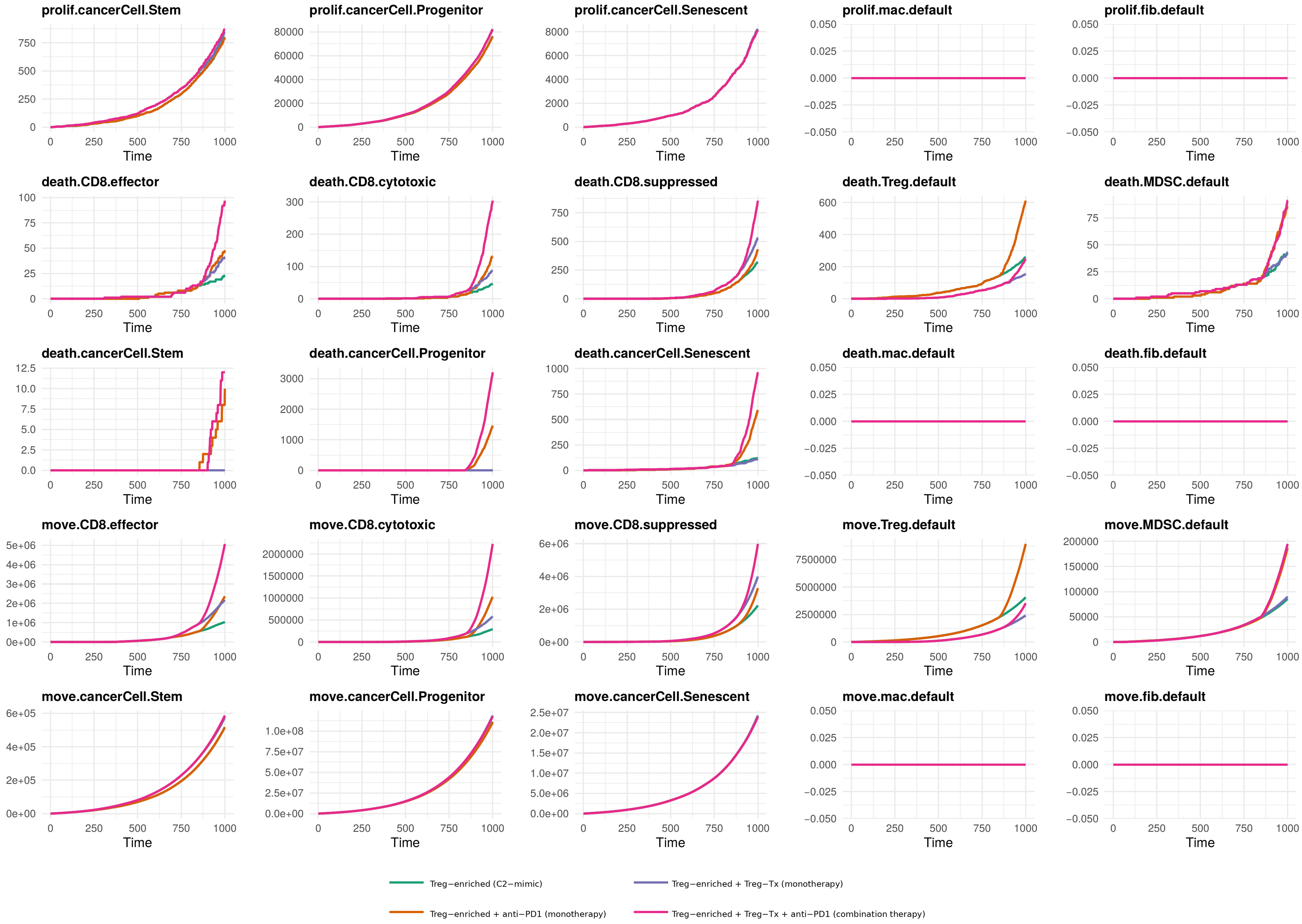
prolif.cancerCell.Stem
prolif.cancerCell.Progenitor
prolif.cancerCell.Senescent
prolif.mac.default
prolif.fib.default
death.CD8.effector
death.CD8.cytotoxic
death.CD8.suppressed
death.Treg.default
death.MDSC.default
death.cancerCell.Stem
death.cancerCell.Progenitor
death.cancerCell.Senescent
death.mac.default
death.fib.default
move.CD8.effector
move.CD8.cytotoxic
move.CD8.suppressed
move.Treg.default
move.MDSC.default
move.cancerCell.Stem
move.cancerCell.Progenitor
move.cancerCell.Senescent
move.mac.default
move.fib.default
Time
Treg−enriched (C2−mimic)
Treg−enriched + Treg−Tx (monotherapy)
Treg−enriched + anti−PD1 (monotherapy)
Treg−enriched + Treg−Tx + anti−PD1 (combination therapy)

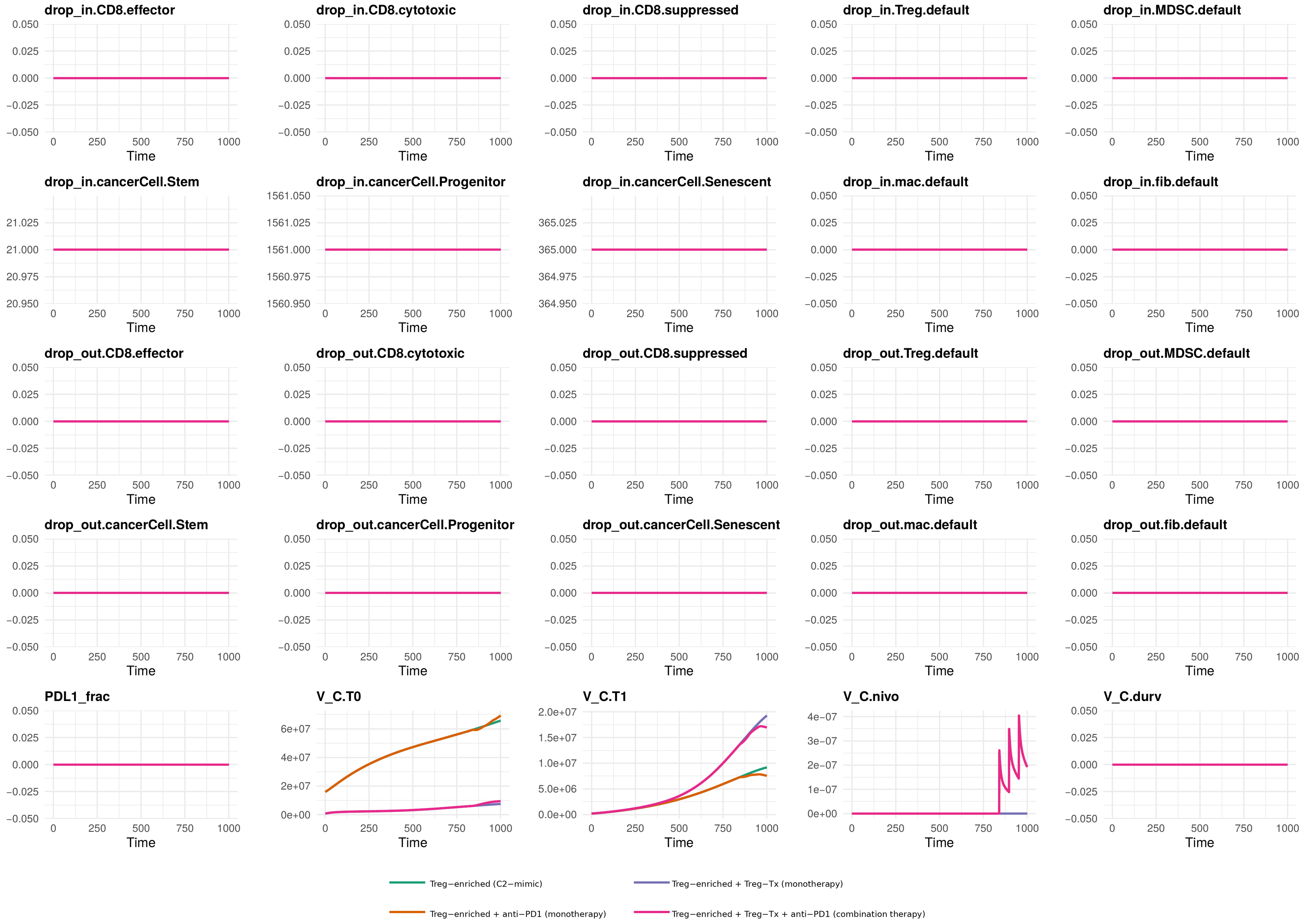
drop_in.CD8.effector
drop_in.CD8.cytotoxic
drop_in.CD8.suppressed
drop_in.Treg.default
drop_in.MDSC.default
drop_in.cancerCell.Stem
drop_in.cancerCell.Progenitor
drop_in.cancerCell.Senescent
drop_in.mac.default
drop_in.fib.default
drop_out.CD8.effector
drop_out.CD8.cytotoxic
drop_out.CD8.suppressed
drop_out.Treg.default
drop_out.MDSC.default
drop_out.cancerCell.Stem
drop_out.cancerCell.Progenitor
drop_out.cancerCell.Senescent
drop_out.mac.default
drop_out.fib.default
PDL1_frac
V_C.T0
V_C.T1
V_C.nivo
V_C.durv
Time
Treg−enriched (C2−mimic)
Treg−enriched + Treg−Tx (monotherapy)
Treg−enriched + anti−PD1 (monotherapy)
Treg−enriched + Treg−Tx + anti−PD1 (combination therapy)

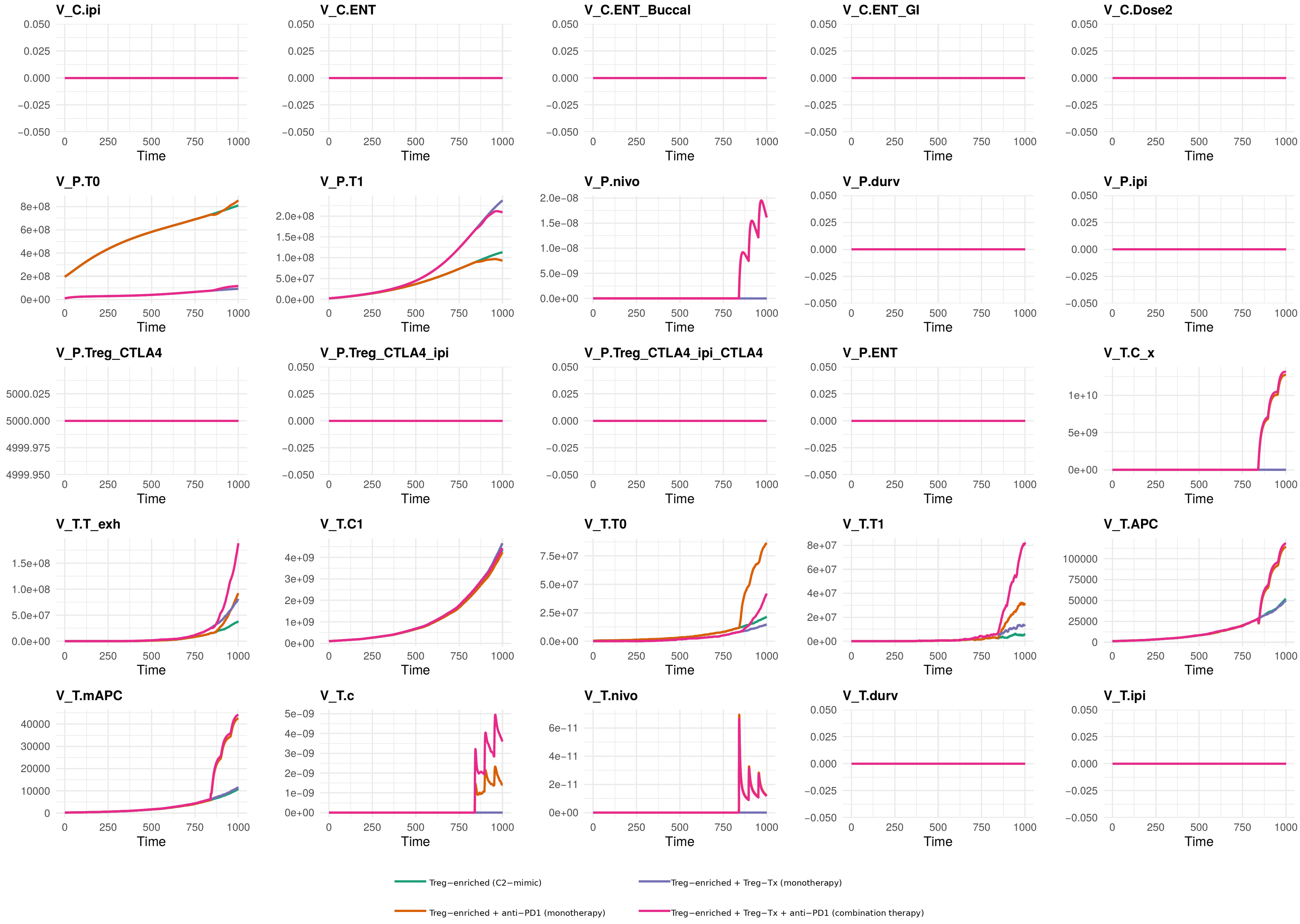
V_C.ipi
V_C.ENT
V_C.ENT_Buccal
V_C.ENT_GI
V_C.Dose2
V_P.T0
V_P.T1
V_P.nivo
V_P.durv
V_P.ipi
V_P.Treg_CTLA4
V_P.Treg_CTLA4_ipi
V_P.Treg_CTLA4_ipi_CTLA4
V_P.ENT
V_T.C_x
V_T.T_exh
V_T.C1
V_T.T0
V_T.T1
V_T.APC
V_T.mAPC
V_T.c
V_T.nivo
V_T.durv
V_T.ipi
Time
Treg−enriched (C2−mimic)
Treg−enriched + Treg−Tx (monotherapy)
Treg−enriched + anti−PD1 (monotherapy)
Treg−enriched + Treg−Tx + anti−PD1 (combination therapy)

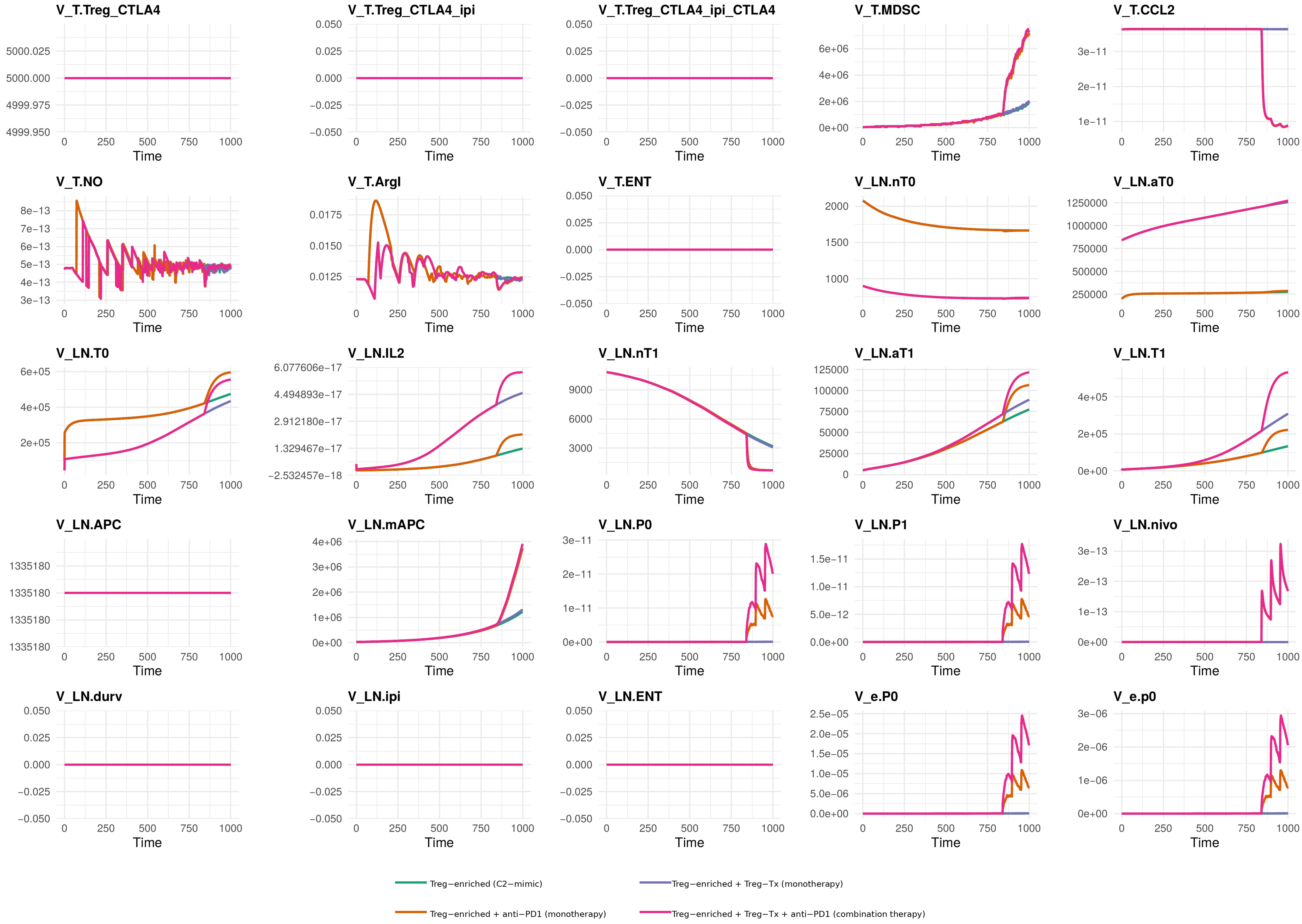
V_T.Treg_CTLA4
V_T.Treg_CTLA4_ipi
V_T.Treg_CTLA4_ipi_CTLA4
V_T.MDSC
V_T.CCL2
V_T.NO
V_T.ArgI
V_T.ENT
V_LN.nT0
V_LN.aT0
V_LN.T0
V_LN.IL2
V_LN.nT1
V_LN.aT1
V_LN.T1
V_LN.APC
V_LN.mAPC
V_LN.P0
V_LN.P1
V_LN.nivo
V_LN.durv
V_LN.ipi
V_LN.ENT
V_e.P0
V_e.p0
Time
Treg−enriched (C2−mimic)
Treg−enriched + Treg−Tx (monotherapy)
Treg−enriched + anti−PD1 (monotherapy)
Treg−enriched + Treg−Tx + anti−PD1 (combination therapy)

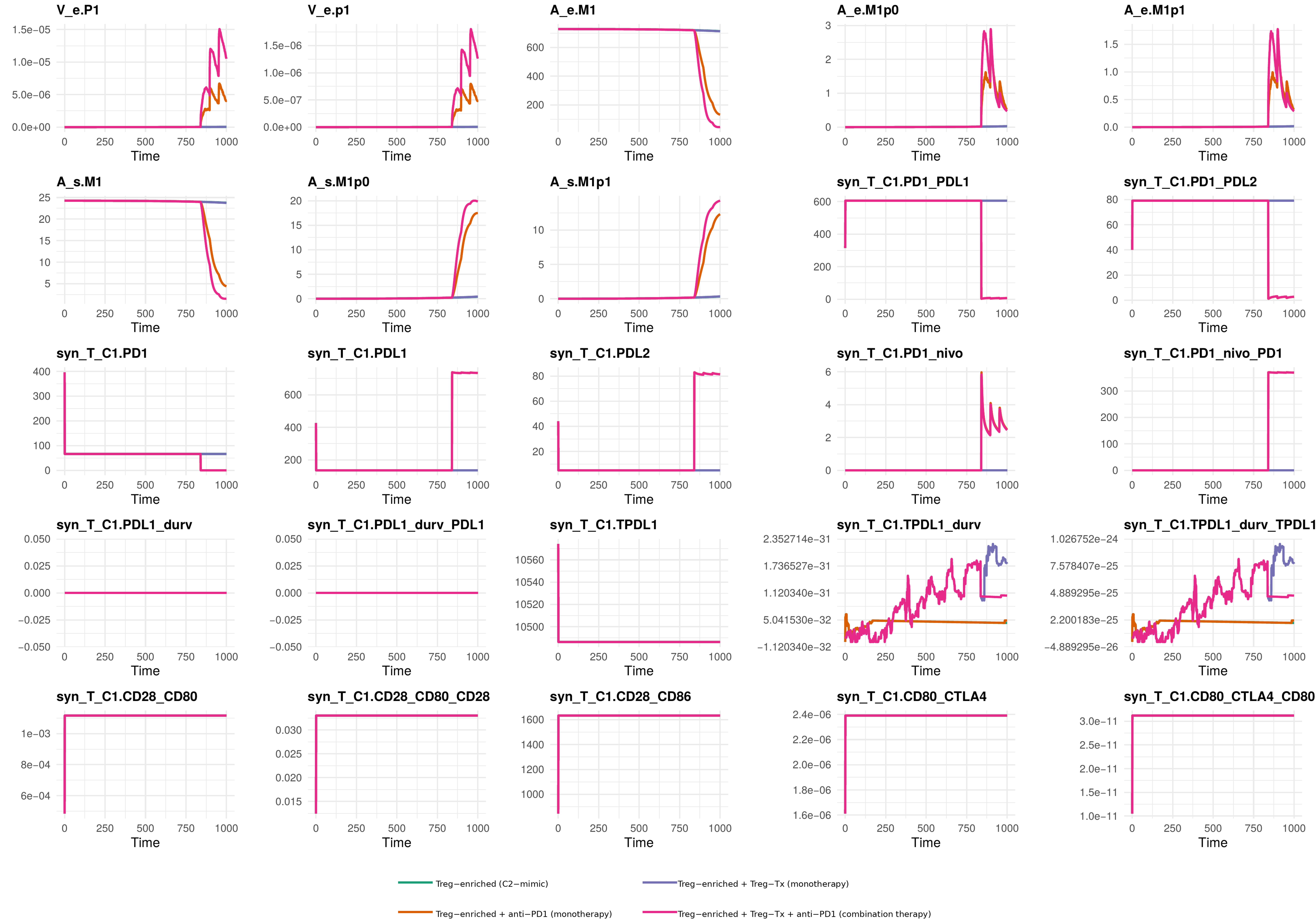
V_e.P1
V_e.p1
A_e.M1
A_e.M1p0
A_e.M1p1
A_s.M1
A_s.M1p0
A_s.M1p1
syn_T_C1.PD1_PDL1
syn_T_C1.PD1_PDL2
syn_T_C1.PD1
syn_T_C1.PDL1
syn_T_C1.PDL2
syn_T_C1.PD1_nivo
syn_T_C1.PD1_nivo_PD1
syn_T_C1.PDL1_durv
syn_T_C1.PDL1_durv_PDL1
syn_T_C1.TPDL1
syn_T_C1.TPDL1_durv
syn_T_C1.TPDL1_durv_TPDL1
syn_T_C1.CD28_CD80
syn_T_C1.CD28_CD80_CD28
syn_T_C1.CD28_CD86
syn_T_C1.CD80_CTLA4
syn_T_C1.CD80_CTLA4_CD80
Time
Treg−enriched (C2−mimic)
Treg−enriched + Treg−Tx (monotherapy)
Treg−enriched + anti−PD1 (monotherapy)
Treg−enriched + Treg−Tx + anti−PD1 (combination therapy)

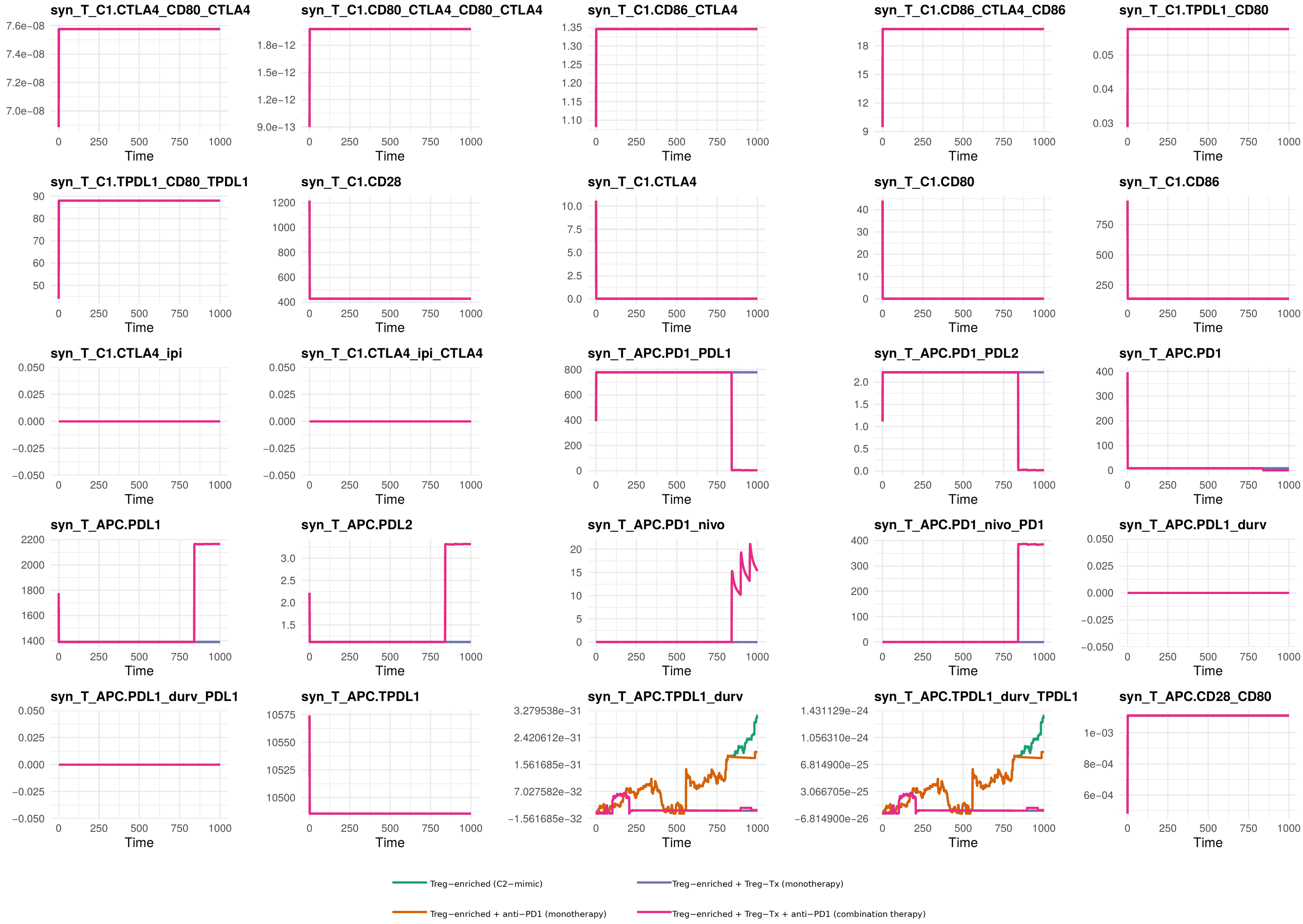
syn_T_C1.CTLA4_CD80_CTLA4
syn_T_C1.CD80_CTLA4_CD80_CTLA4
syn_T_C1.CD86_CTLA4
syn_T_C1.CD86_CTLA4_CD86
syn_T_C1.TPDL1_CD80
syn_T_C1.TPDL1_CD80_TPDL1
syn_T_C1.CD28
syn_T_C1.CTLA4
syn_T_C1.CD80
syn_T_C1.CD86
syn_T_C1.CTLA4_ipi
syn_T_C1.CTLA4_ipi_CTLA4
syn_T_APC.PD1_PDL1
syn_T_APC.PD1_PDL2
syn_T_APC.PD1
syn_T_APC.PDL1
syn_T_APC.PDL2
syn_T_APC.PD1_nivo
syn_T_APC.PD1_nivo_PD1
syn_T_APC.PDL1_durv
syn_T_APC.PDL1_durv_PDL1
syn_T_APC.TPDL1
syn_T_APC.TPDL1_durv
syn_T_APC.TPDL1_durv_TPDL1
syn_T_APC.CD28_CD80
Time
Treg−enriched (C2−mimic)
Treg−enriched + Treg−Tx (monotherapy)
Treg−enriched + anti−PD1 (monotherapy)
Treg−enriched + Treg−Tx + anti−PD1 (combination therapy)

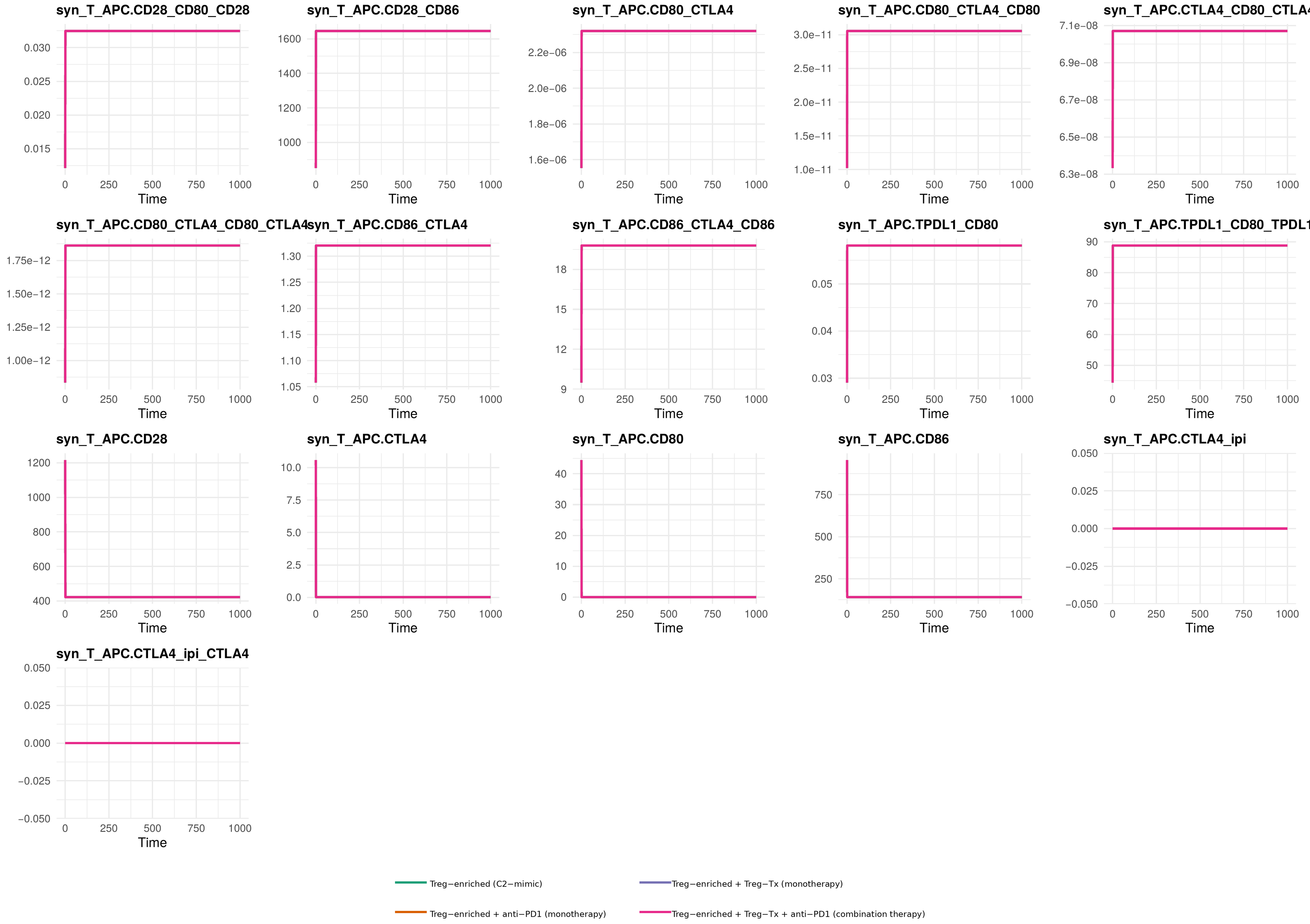
syn_T_APC.CD28_CD80_CD28
syn_T_APC.CD28_CD86
syn_T_APC.CD80_CTLA4
syn_T_APC.CD80_CTLA4_CD80
syn_T_APC.CTLA4_CD80_CTLA
syn_T_APC.CD80_CTLA4_CD80_CTLA4
syn_T_APC.CD86_CTLA4
syn_T_APC.CD86_CTLA4_CD86
syn_T_APC.TPDL1_CD80
syn_T_APC.TPDL1_CD80_TPDL1
syn_T_APC.CD28
syn_T_APC.CTLA4
syn_T_APC.CD80
syn_T_APC.CD86
syn_T_APC.CTLA4_ipi
syn_T_APC.CTLA4_ipi_CTLA4
Time
Treg−enriched (C2−mimic)
Treg−enriched + Treg−Tx (monotherapy)
Treg−enriched + anti−PD1 (monotherapy)
Treg−enriched + Treg−Tx + anti−PD1 (combination therapy)
